# The oral and lower airway microbiota and coronary heart disease in COPD patients and controls

**DOI:** 10.1371/journal.pone.0353738

**Published:** 2026-07-16

**Authors:** Christina Due Svendsen, Rune Nielsen, Terje H. Larsen, Karel K. J. Kuiper, Tomas M. Eagan

**Affiliations:** 1 Department of Thoracic Medicine, Haukeland University Hospital, Bergen, Norway; 2 Department of Clinical Science, University of Bergen, Bergen, Norway; 3 Department of Heart Disease, Haukeland University Hospital, Bergen, Norway; 4 Department of Biomedicine, University of Bergen, Bergen, Norway; The University of Kansas Health Systems St. Francis Campus, UNITED STATES OF AMERICA

## Abstract

**Background:**

Chronic obstructive pulmonary disease (COPD) and coronary heart disease (CHD) are major causes of morbidity and mortality, with shared risk factors and often co-occurring. This study investigated the association between both the oral and lower airway microbiome and CHD in healthy controls and COPD patients.

**Methods:**

228 participants from the MicroCOPD study (101 controls and 127 COPD patients) underwent coronary CT angiography to assess calcium score (CaSc) and coronary stenosis. Oral wash (OW) and bronchoalveolar lavage (BAL) samples were collected. Microbial DNA was analyzed using 16S rRNA gene sequencing with the Illumina MiSeq platform. Microbiome composition and diversity were analysed using established pipelines in Quantitative Insights into Microbial Ecology 2 (QIIME 2) and R.

**Results:**

Alpha diversity (Shannon index) differed significantly between COPD patients and controls in OW (p < 0.01), but not BAL. No statistically significant alpha (Shannon or Faith’s PD) diversity differences were found between CHD and non-CHD groups. Beta diversity analysis (Bray-Curtis dissimilarity) revealed no significant differences in microbial composition between CHD and non-CHD groups, both for COPD patients and controls (p > 0.05).

*Firmicutes* dominated across all subgroups, followed by *Bacteroidetes* and *Actinobacteria*. Several taxa were found to be differentially abundant between CHD and non-CHD groups but comprised less than 1% of all taxa.

**Conclusion:**

The microbiome differed between COPD patients and controls, but we could not find evidence that either the oral or lower airway microbiome differed between those with and without coronary heart disease.

## Introduction

Chronic obstructive pulmonary disease (COPD) and coronary heart disease (CHD) are two of the leading causes of morbidity and mortality worldwide. COPD affects approximately 10% of the adult population and is primarily caused by exposure to harmful particles or gases, most notably tobacco smoke [1,2]. COPD is characterized by airflow limitation and persistent respiratory symptoms such as dyspnea and cough with phlegm, and COPD patients frequently experience exacerbations. CHD affects 1–6% of the adult population in Europe [3] and the United States [4]. CHD results from the buildup of atherosclerotic plaques in the coronary arteries, leading to reduced blood flow to the myocardium and is a major contributor to cardiovascular-related deaths globally [5]. Individuals suffering from COPD are at a significantly higher risk of developing cardiovascular diseases, including CHD [6]. Shared risk factors such as smoking and senescence contribute to the coexistence of these conditions [7,8]. Systemic inflammation contributes to the pathogenesis of both diseases and could be a mechanism for the increased prevalence of CHD in COPD, in addition to the effects of smoking and senescence. The airway inflammation in COPD might spill over in the systemic circulation, where the chronic inflammatory process can lead to endothelial dysfunction, atherosclerosis, and increased risk of CHD [7].

The airway microbiota is a component the local mucosal immune environment must regulate to maintain airway homeostasis. Dysbiosis, or an imbalance in this microbial community, has been observed in COPD patients, with an increase in pathogenic bacteria such as *Haemophilus influenzae* and *Moraxella catarrhalis* [9–11]. This dysbiosis is suggested to contribute to chronic airway inflammation and exacerbations in COPD [12], although longitudinal evidence remains limited [13].

Dysbiosis in the periodontal, oral, and gut microbiomes have all been suggested to influence cardiovascular health [14–16]. To our knowledge, no study has yet examined the association between the lower airway microbiome and CHD, even though the pulmonary circulation likely exposes the cardiac endothelium to a range of metabolites, cytokines, and microbial constituents.

We hypothesized that the oral and lower airway microbiome both could be associated with CHD. The aim of the current study was to compare the oral and lower airway bacterial microbiota in subjects with and without CHD for both healthy controls and COPD patients.

## Methods

### Study population

Participants were recruited from the MicroCOPD study, conducted 2012–2015 in Bergen, Norway. The MicroCOPD study included 103 non-COPD controls and 130 COPD patients who underwent at least one bronchoscopy for sampling of the lower airway microbiome. Patients and controls were recruited from previous patient control studies, through media attention, and our outpatient clinic. The detailed study protocol and some of the results are published [10,13,17].

COPD was defined as chronic airway obstruction characterized by a post-bronchodilator forced expiratory volume in 1 second (FEV_1)_/forced vital capacity (FVC) < 0.7 on standardized spirometry, in addition to the presence of respiratory symptoms, age over 40 years, and a tobacco smoking history of more than 10 pack-years. Severity of airway obstruction was assessed by use of the Global Initiative for Chronic Obstructive Lung Disease (GOLD) criteria [18]. Control subjects were defined as without COPD diagnosis or any other pulmonary diseases.

Inclusion criteria for both groups were age ≥ 40 years, clinical stability at the time of bronchoscopy, and no use of antibiotics within the preceding 14 days. Exclusion criteria for both groups were antibiotic use within 14 days prior to bronchoscopy, unstable coronary heart disease, atrial fibrillation, recent thromboembolic disease, use of anticoagulants other than acetylsalicylic acid, hypercapnia or hypoxemia, and known allergy to lidocaine, alfentanil, or contrast fluids. For COPD patients, exacerbations or treatment for exacerbations within the last 14 days were cause for postponing inclusion. The 14-day antibiotic washout period was selected to balance microbiome integrity against practical inclusion of patients with frequent exacerbations. In sensitivity analyses from the MicroCOPD cohort, 3% of participants had received antibiotics within four weeks and 11% within three months prior to bronchoscopy [10,19].

**Fig 1:**
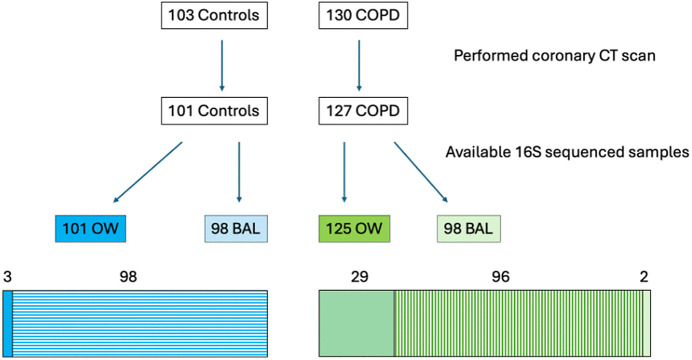
Flow chart over study participants and sample availability.

As part of a larger cross-sectional assessment of COPD and CHD [20], the MicroCOPD participants were also offered a combined pulmonary and coronary angiography CT scan. The current study sample includes those participants who had microbial sampling from either the oral cavity or lower airway by bronchoscopy and had a valid coronary CT scan; in total 101 controls and 127 COPD patients (Fig 1).

### Ethics approval and consent to participate

The Norwegian regional medical ethics committee (REK-Nord) approved the project (REK numbers 2010/2015 & 2011/1307), and the study was performed in accordance with the Declaration of Helsinki. All participants received oral and written information prior to inclusion, and all participants gave written informed consent.

### Data collection

Participants were examined at the outpatient clinic at the Department of Thoracic Medicine, Haukeland University Hospital. A study physician performed a structured interview including smoking history, screening for respiratory symptoms, all current medication use, previous and ongoing medical issues, and exacerbation history for COPD patients. Lung function was measured prior to bronchoscopy, after inhalation of 0.4 mg salbutamol to measure post-bronchodilator FEV_1_ and FVC.

All bronchoscopies were performed without other indication than as part of the study sampling. Oral wash (OW) was sampled by gargling 10 mL sterile saline for 1 minute before bronchoscopy. The bronchoscopy procedure included oral topical anesthesia and light general anesthesia with alfentanil before per-procedural anesthesia was administered via the bronchoscope. Bronchoscopy was performed with the participant in the supine position through oral access, and no suctioning was performed prior to entering trachea. Bronchoalveolar lavage (BAL) was only performed if FEV_1_ was > 30% predicted or > 1 L. BAL sampling was performed with 2 x 50 mL phosphate buffered saline (PBS) via a protected sterile catheter (Plastimed Combicath) with a sealed wax tip, from the right middle lobe. PBS was taken from a new sterile bottle each bronchoscopy day, and a 2 mL sample taken directly from the pristine bottle served as a negative control sample for each participant.

The complete protocol for the coronary computed tomography angiography (CCTA) has been published [20]. Briefly, the CCTA was used for calculation of Agatstons coronary artery calcium score (CaSc), and a CaSc > 100 was considered an indicator of high risk for coronary heart disease [21]. In addition, the CCTA was evaluated for presence of significant coronary artery stenosis defined as a lumen reduction > 50% as judged by a cardiac radiologist and interventional cardiologist.

### Laboratory handling

The detailed laboratory protocol on DNA extraction, PCR, and Illumina sequencing is published [22]. The V3-V4 region of the 16S rRNA gene was amplified using specific primers in the PCR setup. The purified PCR products were then subjected to paired-end, high-throughput sequencing using the Illumina MiSeq platform.

### Bioinformatics and statistics

Quantitative Insights into Microbial Ecology 2 (QIIME 2, version 2022.8) was used as the bioinformatics platform, with appropriate R packages for additional analysis [23,24].

Demultiplexed paired-end sequences were trimmed, denoised, and filtered to remove chimeras using the DADA2 plugin and VSEARCH to generate amplicon sequencing variant (ASV) tables [25,26]. The package Decontam in R [27] was used to remove sequences identified in the negative controls, which were typically introduced in the DNA extraction kit [28].

Taxonomic classification was performed using the Human Oral Microbiome Database (HOMD) [29], with additional sequence verification using the Basic Local Alignment Search Tool (BLAST) [25]. Non-bacterial sequences were removed, and a phylogenetic tree was constructed using FastTree [30]. After quality filtering, the final feature table contained 419 samples and 491 features, with a total of 7 701 954 sequences. Sequencing depth per sample ranged from 8 to 88 581 reads (median 14 175, mean 14 382). Across all features, total read counts ranged from 19 to 1 321 842 (median 1 919 reads per feature).

To assess potential confounding by medication use, sensitivity analyses were performed for alpha diversity, beta diversity, and differential abundance. Each medication class (long-acting muscarinic antagonists (LAMA), long-acting beta agonists (LABA), inhaled corticosteroids (ICS), ACE inhibitors, angiotensin II receptor blockers (ARB), acetylsalicylic acid, statins, and proton pump inhibitors (PPI)), was added individually as a covariate to the base model (CaSc + age + sex + smoking).

### Alpha diversity

Alpha diversity was quantified using Shannon diversity index (accounting for richness and evenness) and Faith’s phylogenetic diversity (incorporating evolutionary relationships between taxa). Differences in alpha diversity between groups (healthy controls, COPD patients, low or high CaSc, and presence of coronary artery stenosis) were assessed using Wilcoxon rank-sum test for unadjusted pairwise comparisons. For adjusted analysis accounting for potential confounders such as age, sex, and smoking status, we employed generalized linear models with geometric mean (GM) ratio of Shannon diversity or Faith’s PD as the outcome variable.

### Beta diversity

Beta diversity was assessed using permutational multivariate analysis of variance (PERMANOVA) with the vegan package in R, testing for differences in overall bacterial community composition between groups while adjusting for age, sex, and smoking status. Principal Coordinates Analysis (PCoA) was performed on the Bray-Curtis dissimilarity matrix to reduce the high-dimensional data into two-dimensional space, enabling visualization of between-sample relationships and community clustering by group.

### Taxonomic composition and differential abundance

Bacterial taxa were analysed at the levels of phylum and genus. Relative abundance was calculated as the proportion of reads assigned to each taxon relative to total reads per sample. Differences in taxon abundance between groups were identified using analysis of compositions of microbes with bias correction 2 (ANCOM-BC2), a compositionally aware method that accounts for differential sequencing depth, library composition bias, and sparse count data. ANCOM-BC2 provides W-statistics and adjusted p-values for each taxon-group comparison, taxa with adjusted p < 0.05 were considered significantly differentially abundant.

## Results

The baseline characteristics of the study participants are presented in Table 1. Sex and age did not differ between controls and COPD patients, but the COPD patients had smoked more, had lower lung function, and a higher share of the COPD patients had CaSc > 100. More than twice as many COPD patients had a significant coronary stenosis in terms of lumen reduction compared to the control subjects, although the difference in prevalence was not statistically significant.

**Table 1:** Baseline characteristics for the MicroCOPD study participants with a coronary CT scan.

	Controls	COPD	
	n=101 (44.3%)	n=127 (55.7%)	p*
*Sex, n (%)*			0.99
Women	43 (44.3)	54 (44.3)	
Men	58 (55.7)	73 (55.7)	
*Age, mean (SD)*	65.8 (7.9)	67.4 (7.3)	0.11
*Smoking, n (%)*			<0.01
Never	16 (15.8)	0	
Ex	57 (56.4)	89 (70.1)	
Daily	28 (27.7)	38 (29.9)	
*Pack years, mean (SD)*	21.9 (13.5)	34.3 (31.0)	<0.01
*Lung function*			
FEV_1_ in percent predicted, mean (SD)	104.2 (12.5)	56.6 (19.6)	<0.01
FVC in percent predicted, mean (SD)	112.2 (13.2)	94.4 (18.0)	<0.01
*Calcium Score (CaSc), n (%)*			<0.01
< 100	70 (69.3)	58 (46.6)	
≥ 100	31 (30.7)	67 (53.6)	
*Significant coronary stenosis, n (%)*			0.18
No	79 (95.2)	89 (89.0)	
Yes	4 (4.8)	11 (11.0)	
*Statin use, n (%)*			0.15
No	80 (79.2)	90 (70.9)	
Yes	21 (20.8)	37 (29.1)	

* Chi square test for sex, calcium score and statin use, ANOVA for age, pack years and lung function, and Fisher’s exact test for smoking and coronary stenosis

### Diversity

A total of 491 ASVs were identified across all samples. OW samples demonstrated high concordance between COPD and control subjects, with 412 of 445 COPD-detected ASVs (92.6%) also present in controls. Only 33 ASVs (7.4%) were unique to COPD patients. Controls had similarly low uniqueness, with 30 OW-specific ASVs (6.8%). BAL samples showed distinct patterns, with COPD patients harboring 94 unique ASVs (23.6% of COPD ASVs) and controls harboring 47 unique ASVs (13.4% of control ASVs), indicating sample site-dependent microbiota composition. OW samples showed high ASV overlap between COPD patients and controls (80–86%), whereas BAL samples were more distinct, with lower overlap (62–79%) and more ASVs unique to each group, particularly in the high CaSc category.

**Fig 2:**
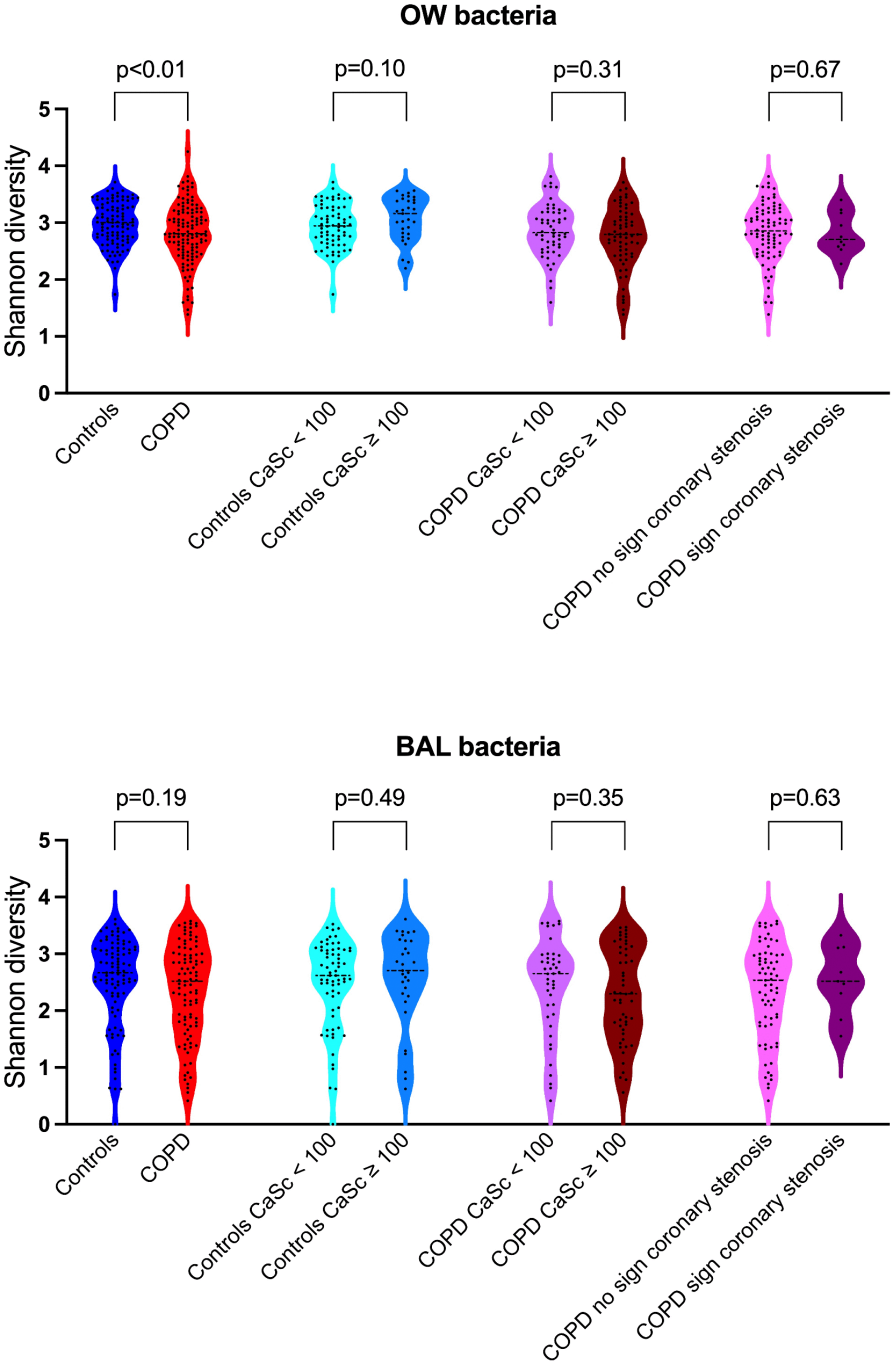
Shannon alpha diversity index in OW and BAL samples from controls and COPD patients with and without CT indices of coronary heart disease.

Alpha diversity was assessed with both non-phylogenetic (Shannon index) and phylogenetic (Faith’s PD) indices. COPD patients had significantly (p < 0.01) lower alpha diversity in the OW samples by the Shannon index compared to controls (Fig 2), and nearly significantly lower by Faith’s PD (p = 0.06, S1 Fig). However, no differences were observed between participants with or without signs of CHD (p > 0.05 all comparisons), for either Shannon or Faith’s PD (Fig 2 & S1 Fig).

In multivariate models adjusting for age, sex, and smoking, the same pattern was apparent with no difference in alpha diversity between subjects with and without CHD, neither in healthy controls nor in COPD patients (Table 2).

**Table 2:** Adjusted* alpha diversity (Shannon index and Faith’s PD index) of bacterial taxa in oral wash (OW) and bronchoalveolar lavage (BAL) between those with and without CT indices of coronary disease, in healthy controls and in COPD patients.

	n	Shannon			Faith’s PD		
		GM	95% CI	p	GM	95% CI	p
*Controls OW*							
Low CaSc	70	1			1		
High CaSc	31	1.06	0.997 - 1.14	0.06	1.10	0.97 - 1.25	0.13
*COPD OW*							
Low CaSc	56	1			1		
High CaSc	65	0.99	0.91 - 1.07	0.70	1.01	0.87 - 1.17	0.18
*Controls BAL*							
Low CaSc	67	1			1		
High CaSc	31	0.96	0.79-1.17	0.71	1.06	0.81 - 1.38	0.69
*COPD BAL*							
Low CaSc	43	1			1		
High CaSc	52	0.89	0.72 - 1.10	0.28	0.83	0.64 - 1.09	0.19
*COPD OW*							
No stenosis	86	1			1		
Stenosis	10	1.00	0.88 - 1.14	0.96	1.03	0.81 - 1.31	0.81
*COPD BAL*							
No stenosis	71	1			1		
Stenosis	9	1.08	0.77 - 1.52	0.64	1.09	0.72 - 1.65	0.69

* Adjusted for age, sex and smoking.

Examining beta diversity (Bray-Curtis dissimilarity), no significant differences between samples were observed between controls and COPD patients in either OW or BAL samples when stratified by CaSc, or presence of significant coronary stenosis in COPD patients. Across all subgroups, R^2^-values were low, indicating that only a small proportion of the variance in microbial composition was explained by these groupings. The findings remained consistent after adjusting for age, sex, and smoking status (S1 Table). Principal Coordinate Analysis (PCoA) plots further illustrated the lack of distinct clustering between groups (S2 Fig).

### Taxonomy

**Fig 3:**
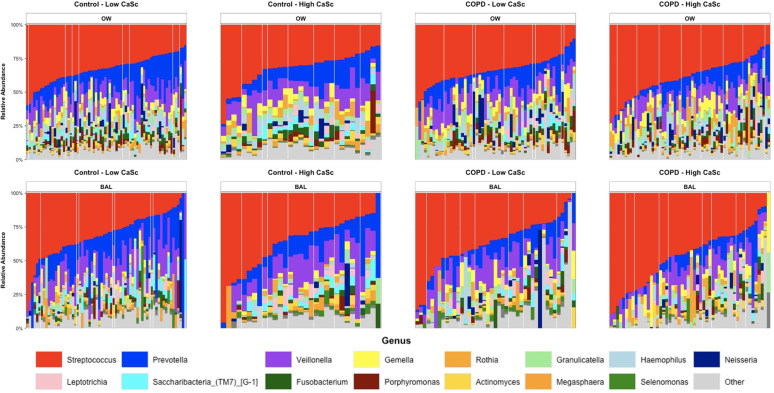
Bacterial genera composition stratified by sample type (OW and BAL), diagnosis (COPD and controls), and coronary artery calcium score (low and high CaSc). Each vertical bar represents one participant ordered from left to right by decreasing abundance sorted by Streptococcus.

*Firmicutes* was identified as the most abundant bacterial phylum across all subgroups, regardless of CaSc or coronary artery stenoses. More than 50% of phyla in OW or BAL samples belonged to *Firmicutes*; *Bacteroidetes* being the second most common, and *Actinobacteria* the third. The relative abundance of the 15 most common bacterial genera in OW and BAL samples for controls and COPD patients are presented in Fig 3, stratified by high (CaSc > 100) or low calcium score (CaSc < 100). Visually, streptococci were the most common genus, followed by Prevotella and Veilonella (Fig 3).

Differential abundance testing between participants with and without signs of CHD for bacterial taxa is presented in Table 3. The first two columns show the mean relative abundance (%) of the taxon in question for those with low or high calcium scores, or no or significant stenosis in COPD patients. Only statistically significant (q < 0.05) taxa are named. The log fold change (lfc) values with corresponding q-values are presented without and with adjustment for age, sex, and smoking. On the phylum level, *Bacteriodetes* on OW were relatively more abundant in controls with low calcium score (21.8%) versus in controls with high calcium score (18.4%). There were zero significantly different phyla between COPD patients with and without coronary stenosis. For genera, only nine were found significantly different between the various groups compared, out of a potential 69. Those nine were all of low relative prevalence, less than 0.8% for all taxa (Table 3).

**Table 3:** Differentially abundant phyla and genera in OW and BAL between those with and without CT indices of coronary disease, both in healthy controls and COPD patients.

*phyla*			Unadjusted		Adjusted*	
	% low CaSc	% high CaSc	lfc high CaSc	q	lfc high CaSc	q
Controls OW						
*Bacteroidetes*	21.80	18.40	−0,69	0.036		
Controls BAL						
*Absconditabacteria_(SR1)*	0.17	0.04	−1.32	0.013		
COPD OW						
*Absconditabacteria_(SR1)*	0.11	0.06	−0.74	0.017		
*Spirochaetes*	0.09	0.12			0.94	0.007
COPD BAL						
*Synergistetes*	0.02	0.005	−1.00	0.011		
***genera***			**Unadjusted**		Adjusted*	
	**% low CaSc**	**% high CaSc**	**lfc high CaSc**	**q**	**lfc high CaSc**	**q**
Controls OW						
*Aggregatibacter*	0.10	0,05	−0.93	0.030		
*Lactobacillus*	0	0.70			1.70	0.005
Controls BAL						
*Stomatobaculum*	0.3	0.60	0.83	0.022		
COPD OW						
*Corynebacterium*	0.01	0.09	0.83	0.002		
COPD BAL						
*Bacteroidales_[G-2]*	0.10	0.10	−1.06	0.003	−1.59	0.040
*Bergeyella*	0.0004	0.20	0.72	0.011		
*Ruminococcaceae_[G-1]*	0.10	0.20			−1.22	0.012
			**Unadjusted**		Adjusted*	
	**% no stenosis**	**% significant stenosis**	**lfc significant stenosis**	**q**	**lfc significant stenosis**	**q**
COPD OW						
*Lactobacillus*	0.30	0.70	−0.76	0.003	2.67	0.015
COPD BAL						
*Tannerella*	0.22	0.03			−2.52	0.041

* Adjusted for age, sex, and smoking.

To address the potential influence of medication use on microbiome composition, sensitivity analyses were performed, where each medication class was added individually as a covariate to the base model. The analyses confirmed the primary finding of no significant association between the airway microbiome and CaSc, was robust across all medication-adjusted models for both alpha and beta diversity (S2 and S3 Tables). The enrichment of oral Lactobacillus in Controls with CaSc > 100 remained significant after adjustment for most medication classes, with stable log-fold change estimates across all models (S4 and S5 Tables).

## Discussion

This study investigated the associations between the airway microbiota and CT angiography defined CHD in controls and COPD patients. In general, neither the oral nor the lower airway microbiome were associated with coronary heart disease in a convincing way in neither controls with no known lung disease nor in COPD patients. For neither alpha nor beta diversity could we find differences in the oral and lower airways microbiome in participants with and without CHD. For taxonomy, the few statistically significantly different genera were of low abundance, and without known pathogenic potential in CHD.

An association between periodontal disease and CHD has long been acknowledged, but evidence for a causal relationship has been lacking [31]. Previous studies on the general oral microbiome have suggested possible oral-cardiovascular associations. Tonelli et al reported that dysbiosis in the oral microbiome could be linked to CHD [32], and bacterial DNA presence in atherosclerotic plaque samples has been found to concordantly reflect oral cavity abundance as shown by Jonsson et al [33]. Koren et al [15] demonstrated that the microbial communities present in atherosclerotic plaques were similar to those found in the oral cavity, suggesting the mouth as a source for bacteria associated with plaque formation.

Several potentially causal mechanisms may link the oral and lower airways microbiome to CHD. Oral bacteria can reach the circulation via transient bacteremia from routine activities such as mastication and tooth brushing, and microbial degradation products such as lipopolysaccharides may promote low-grade systemic inflammation and endothelial activation [34]. The present finding of elevated Lactobacillus in controls with CaSc > 100 adds to this literature and points to additional mechanisms. Lactobacillus species are documented producers of GABA via glutamate decarboxylase activity [35], a metabolite with antihypertensive properties, and probiotic supplementation has been shown to lower cholesterol levels by modulating bile acid metabolism and cholesterol absorption [36]. These observations are consistent with data from the large population-based SCAPIS cohort, which linked gut and oral microbial taxa to CT-based coronary atherosclerosis measures [37]. However, whether the Lactobacillus enrichment observed here represents a cause, consequence, or shared determinant of coronary calcium deposition, cannot be established. Although these are attractive hypotheses, there are arguably too few studies performed to date to draw any conclusions.

To our knowledge, this the first study to investigate the potential relationship between the lower airway microbiome and CHD. We examined this relationship both in controls without known lung disease, and in patients with COPD. We collected sterile samples from both the oral cavity and the lower airway via bronchoscopy and assessed CHD objectively using CT angiography. Although no clear association between the oral and lower airway microbiome and CHD could be found, our findings provide a characterization of the microbial composition in clinically relevant subgroups, which may serve as a reference for future research.

In our study, COPD patients showed reduced alpha diversity compared with controls, consistent with a less resilient microbial ecosystem and previous COPD findings. Community composition patterns were similar to earlier reports, with *Firmicutes* dominating both oral wash and BAL samples [38]. The high proportion of shared ASVs between OW and BAL supports the concept that the lower airway microbiota largely represents a subset of the oral microbiota, with lower biomass and greater susceptibility to host-driven changes [39–41].

In a previous report from the MicroCOPD study we have shown that there is a dysbiosis in COPD compared with controls without lung disease [10]. This is in line with the theory that a chronic inflammation from systemic immune responses could by linking airway microbiota with the pathogenesis of COPD. Airway dysbiosis may exacerbate chronic inflammation, leading to tissue damage, impaired lung function, and increased susceptibility to infections [10,12]. Persistent inflammation in the lungs can extend beyond the local environment, spilling over into the systemic circulation, consistent with the “spill-over”-hypothesis [42]. If there were an association between the microbiome and CHD, we would therefore expect it to be most visible in patients with COPD, in whom we had a known airways dysbiosis. That none was found even in the COPD patients is a strong indication that neither the oral nor the lower airways microbiome is causally linked with CHD.

However, while we did not detect an association between the oral or lower airways microbiome and coronary heart disease in our study cohort, subtle or cumulative effects should not be excluded without further studies. It is possible that the low microbial biomass present in the oral and lower airways was insufficiently altered to exert a measurable influence on CHD within our study population. The largest bacterial biomass is contained in the gut, and there has been some studies suggesting that the gut microbiome is associated with CHD [43,44]. The gut also has potential for microbial translocation, and it is well known that the gut microbiome can impact systemic inflammation [45]. If the gut microbiome but not the airways microbiome is associated with CHD, if may be because the microbial biomass is much greater than in the airways, implying that the size of the microbial load matters. However, that is only a hypothesis and remains to be confirmed.

Characterization of the functionality of the microbiome is an important research area. 16S sequencing allows characterization of the compositionality but not directly of the functionality. However, indirect inferences of functionality from compositional data is possible though Phylogenetic Investigation of Communities by Reconstruction of Unobserved States (PICRUSt2) [46] functional pathway analysis. Exploratory PICRUSt2 analyses on the current study sample revealed no significant differences in predicted metabolic pathway abundances between participants with elevated CaSc or low CaSc in either OW or BAL samples (S3 and S4 Figs). This could be interpreted as supporting the absence of a functional microbiome signal associated with coronary calcium score, however, given the inherent limitations of 16S-based functional inference at genus level, we advise caution in interpretation.

There are some methodological issues to acknowledge. First, the cross-sectional study design makes causal inferences impossible. Second, CaSc is a surrogate marker for CHD. However, we also report on significant coronary stenosis from the CT scans, supporting our findings using CaSc. Third, one might think medication use could impact the microbiome by itself. We examined if inclusion to any multivariable model would impact diversity or taxonomic composition of the use of COPD medications like long-acting β2-agonists, long-acting muscarinic antagonists, and inhaled corticosteroids, as well as for all participants medications for hypertension, statins, proton pump inhibitors and platelet inhibitors. For neither diversity nor differential abundance analyses did medication use by our participants impact the results. Fourth, the use of 16S rRNA sequencing provides information at the genus level but restricts species-level evaluation. Fifth, we stress that the oral microbiome is not equivalent to the upper airways microbiome, and is influenced by many factors including diet, oral hygiene, and gum health. Nevertheless, the oral cavity is the primary reservoir seeding the lower airways via microaspiration and thus an important factor shaping the lower airway microbiome. Finally, BAL sampling was consistently performed from the right middle lobe for all patients, which does not account for potential geographical variation of the airway microbiota within the lungs.

In conclusion, this study compared the oral and lower airway microbiota in COPD patients and healthy controls with and without CHD. While COPD patients showed reduced microbial diversity and taxonomic shifts, no significant associations were found between either the oral or lower airway microbiota composition and CHD. COPD patients exhibited higher smoking prevalence, reduced lung function, and greater coronary calcification and stenosis, suggesting that factors beyond airway microbiota may contribute to the increased CHD burden in COPD. Future research should explore the interplay between airway and gut microbiota, systemic inflammation, and cardiovascular health to identify potential therapeutic targets for reducing the dual burden of COPD and CHD.

## Supporting information

S1 Fig Faith’s PD index in OW and BAL samples from controls and COPD patients with and without CT indices of coronary heart disease. (TIFF)

S2 Fig Principal Coordinate Analysis (PCoA) plots of the beta diversity between those with and without CT indices of coronary heart disease in OW and BAL for controls and COPD patients. (TIFF)

S3 Fig PICRUSt2 Functional pathway analysis on OW and BAL samples stratified for low or high CaSc. (TIFF)

S4 Fig PICRUSt2 functional pathway prediction on OW and BAL samples stratified for low or high CaSc in COPD patients and healthy controls. (TIFF)

S1 Table Bacterial beta diversity calculated by Bray-Curtis distance in oral wash (OW) and bronchoalveolar lavage (BAL) for controls and COPD patients. (DOCX)

S2 Table Alpha diversity (Shannon index) for the effect of CaSc when each medication group is added. (DOCX)

S3 Table Beta diversity (Bray-Curtis) for the effect of CaSc when each medication group is added. (DOCX)

S4 Table ANCOM-BC2 medication sensitivity analyses: OW samples – Controls and COPD patients. (DOCX)

S5 Table ANCOM-BC2 medication sensitivity analyses: BAL samples – Controls and COPD patients. (DOCX)
