# Supplementary figures and images for: The oral and lower airway microbiota and coronary heart disease in COPD patients and controls
Source: PLoS One. 2026 Jul 16;21(7):e0353738. doi: 10.1371/journal.pone.0353738 (PMC13374919; doi:10.1371/journal.pone.0353738)

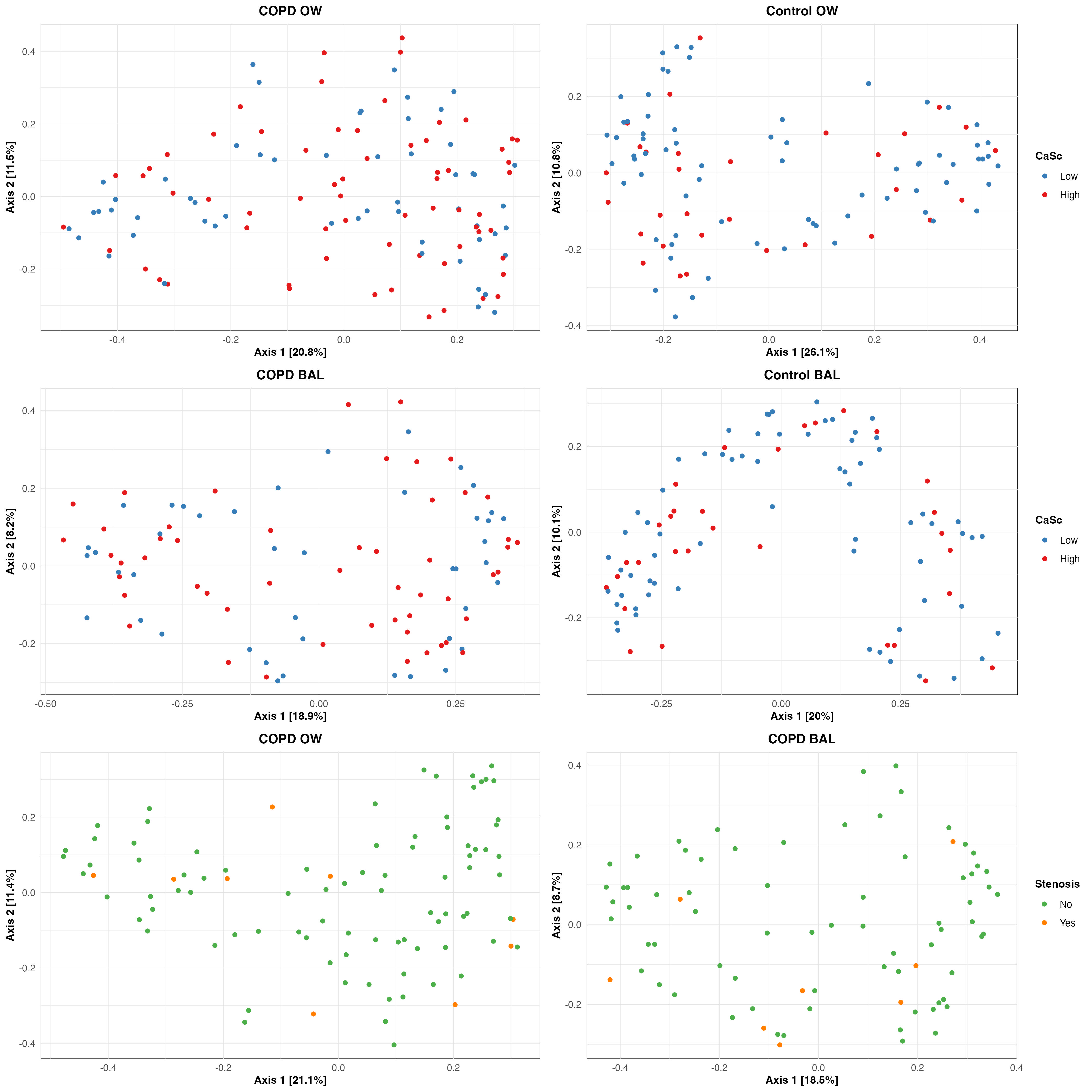

Supplement: S2 Fig — (TIFF) [file pone.0353738.s002.tiff]

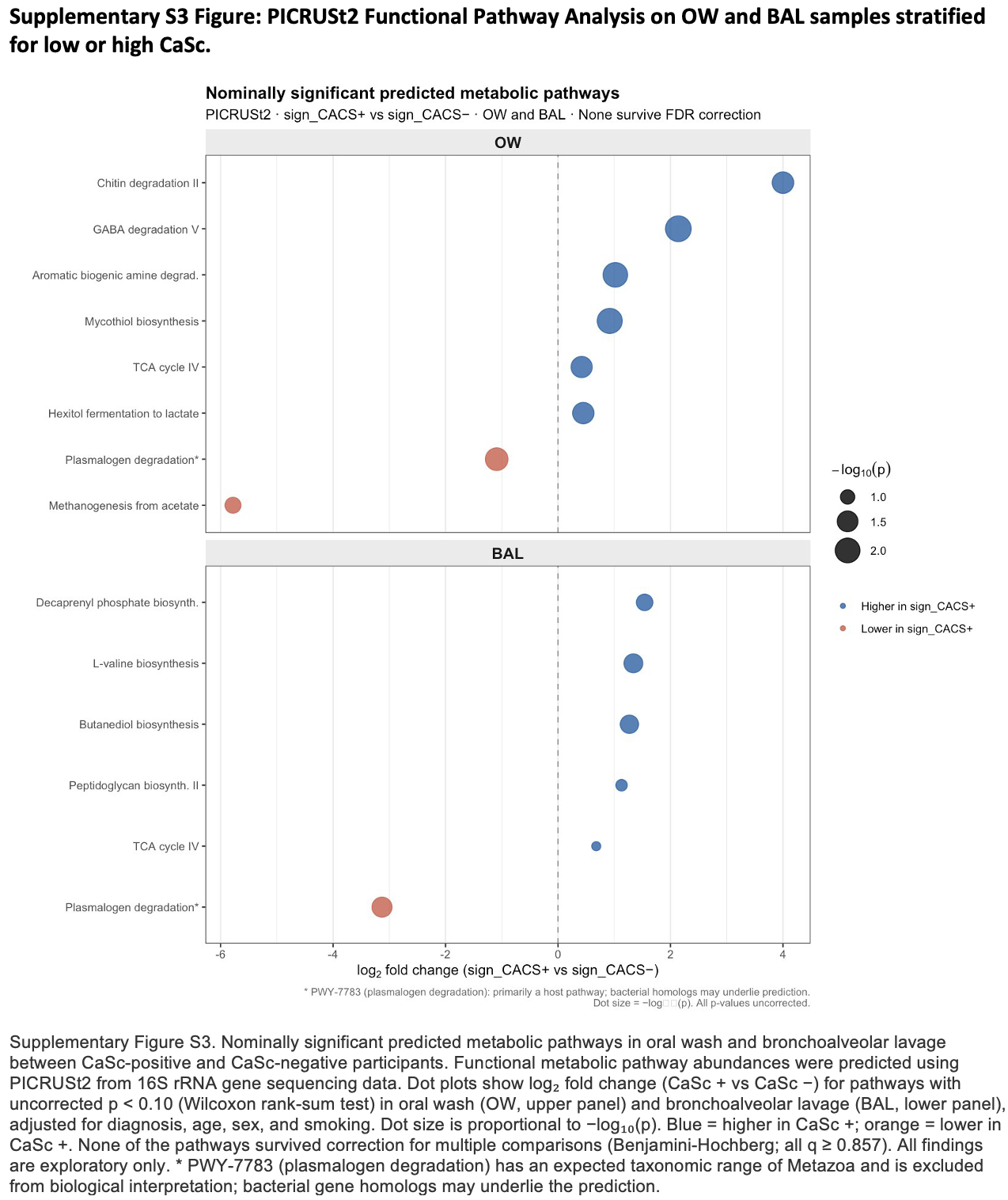

Supplement: S3 Fig — (TIFF) [file pone.0353738.s003.tiff]

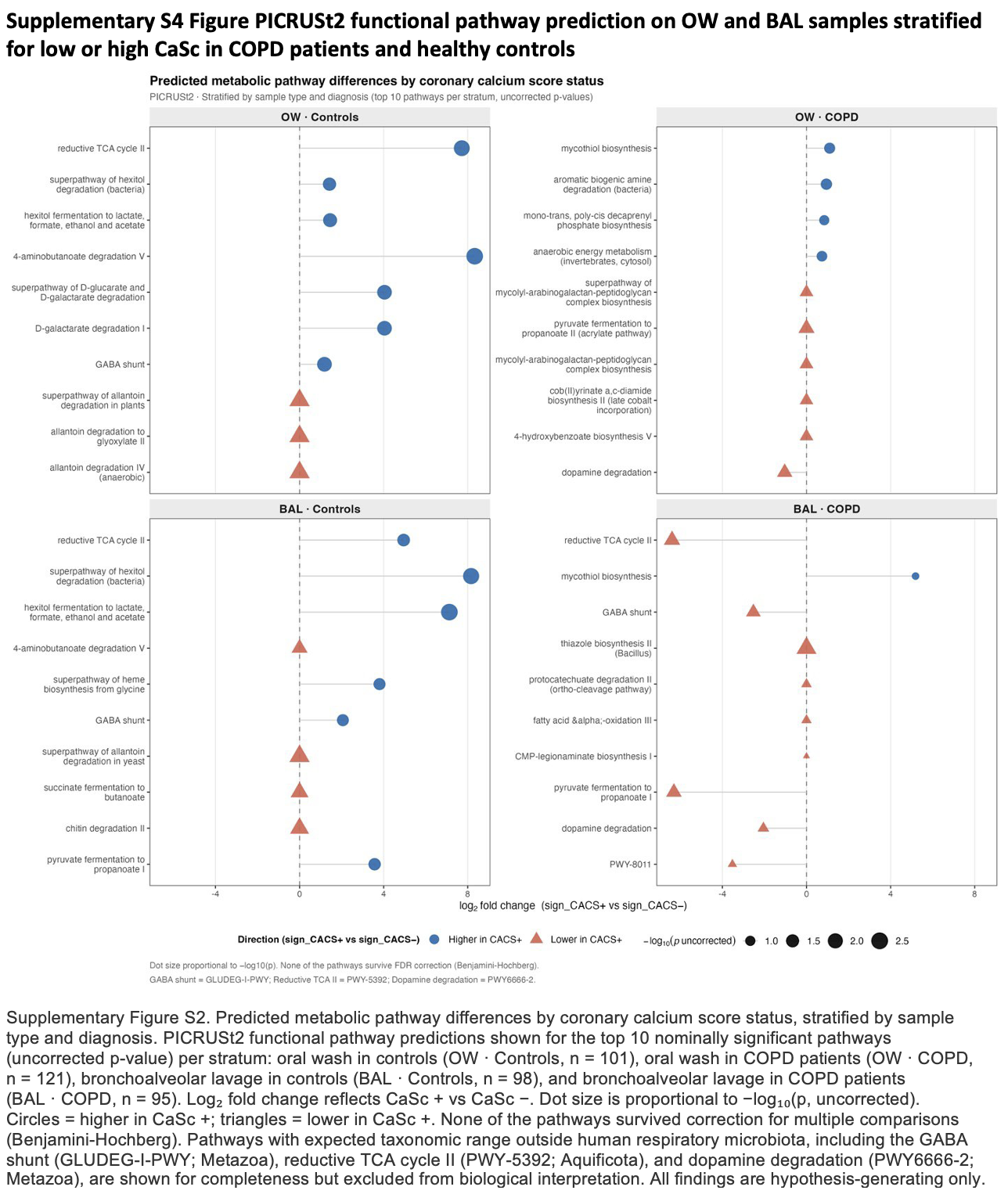

Supplement: S4 Fig — (TIFF) [file pone.0353738.s004.tiff]
