# Supplementary material for: The oral and lower airway microbiota and coronary heart disease in COPD patients and controls
Source: PLoS One. 2026 Jul 16;21(7):e0353738. doi: 10.1371/journal.pone.0353738 (PMC13374919; doi:10.1371/journal.pone.0353738)
Supplement: S1 Table — (DOCX) [file pone.0353738.s005.docx]

**S1 Table. Bacterial beta diversity calculated by Bray-Curtis distance in oral wash (OW) and bronchoalveolar lavage (BAL) for controls and COPD patients**

| Unadjusted beta diversity | | | Adjusted beta diversity | | |
| --- | --- | --- | --- | --- | --- |
| **Variable** | **R²** | **p** | **Variable** | **R²** | **p** |
| Controls OW | | | | | |
| CaSc | 0.011 | 0.30 | CaSc | 0.009 | 0.52 |
|  |  |  | Age | 0.008 | 0.63 |
|  |  |  | Sex | 0.012 | 0.24 |
|  |  |  | Smoking | 0.023 | 0.01 |
| COPD OW | | | | | |
| CaSc | 0.008 | 0.40 | CaSc | 0.007 | 0.61 |
|  |  |  | Age | 0.008 | 0.40 |
|  |  |  | Sex | 0.005 | 0.91 |
|  |  |  | Smoking | 0.012 | 0.11 |
| Controls BAL | | | | | |
| CaSc | 0.008 | 0.79 | CaSc | 0.007 | 0.87 |
|  |  |  | Age | 0.012 | 0.87 |
|  |  |  | Sex | 0.029 | 0.10 |
|  |  |  | Smoking | 0.024 | 0.12 |
| COPD BAL | | | | | |
| CaSc | 0.002 | 0.99 | CaSc | 0.002 | 0.99 |
|  |  |  | Age | 0.005 | 0.24 |
|  |  |  | Sex | 0.013 | 0.17 |
|  |  |  | Smoking | 0.012 | 0.24 |
| COPD OW | | | | | |
| Stenosis | 0.007 | 0.85 | Stenosis | 0.007 | 0.82 |
|  |  |  | Age | 0.016 | 0.09 |
|  |  |  | Sex | 0.006 | 0.94 |
|  |  |  | Smoking | 0.013 | 0.23 |
| COPD BAL | | | | | |
| Stenosis | 0.013 | 0.42 | Stenosis | 0.015 | 0.23 |
|  |  |  | Age | 0.010 | 0.79 |
|  |  |  | Sex | 0.017 | 0.12 |
|  |  |  | Smoking | 0.013 | 0.35 |
|  | | | | | |
