# Supplementary material for: The oral and lower airway microbiota and coronary heart disease in COPD patients and controls
Source: PLoS One. 2026 Jul 16;21(7):e0353738. doi: 10.1371/journal.pone.0353738 (PMC13374919; doi:10.1371/journal.pone.0353738)
Supplement: S2 Table — (DOCX) [file pone.0353738.s006.docx]

**Supplemental S2 Table. Alpha diversity (Shannon index) — linear regression coefficient (β) for calcium score (p-value). Each row shows the effect of calcium score in alpha diversity when the row medication is added as a covariate.**


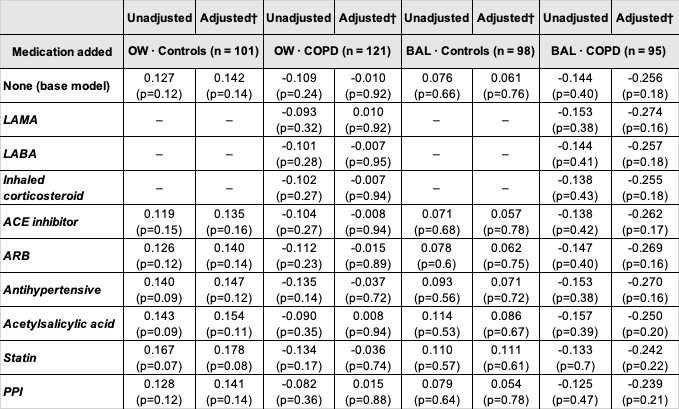


† Adjusted: age, sex, smoking status included as covariates in addition to the listed medication.

– Not applicable: medication class not used in this diagnostic group (e.g., LAMA/LABA/ICS absent in controls).

Base model (yellow): CaSc only (unadjusted) or CaSc + age + sex + smoking (adjusted), without any medication covariate. All p-values are two-sided. None of the CaSc’ effects reached statistical significance in any model.

OW = oral wash; BAL = bronchoalveolar lavage; LAMA = long-acting muscarinic antagonist; LABA = long-acting β2-agonist; ICS = inhaled corticosteroid; ACE = angiotensin-converting enzyme; ARB = angiotensin receptor blocker; PPI = proton pump inhibitor.

**Supplemental S2 Table B. Beta diversity (Bray-Curtis, PERMANOVA) — marginal R² for CaSc (p-value). Each row shows the effect of calcium score when the row medication is added as a covariate.**


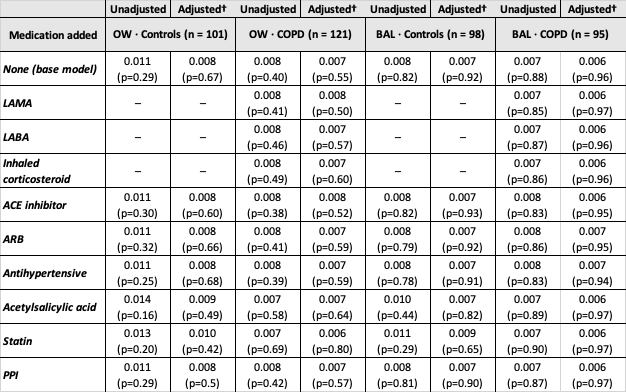


† Adjusted: age, sex, smoking status included as covariates in addition to the listed medication.

– Not applicable: medication class not used in this diagnostic group (e.g., LAMA/LABA/ICS absent in controls).

Base model (yellow): sign_CACS only (unadjusted) or sign_CACS + age + sex + smoking (adjusted), without any medication covariate.

All p-values are two-sided. None of the sign_CACS effects reached statistical significance in any model.

OW = oral wash; BAL = bronchoalveolar lavage; LAMA = long-acting muscarinic antagonist; LABA = long-acting β2-agonist; ICS = inhaled corticosteroid; ACE = angiotensin-converting enzyme; ARB = angiotensin receptor blocker; PPI = proton pump inhibitor.

**Supplemental S2 Table C. ANCOM-BC2 medication sensitivity analyses: OW samples – Controls and COPD patients**

Effect of CaSc on differentially abundant taxa (from Table 3) when individual medication classes are added as covariates. Base model: CaSc + age + sex + smoking. Values shown as log-fold change (q-value, BH-corrected). * q < 0.05.


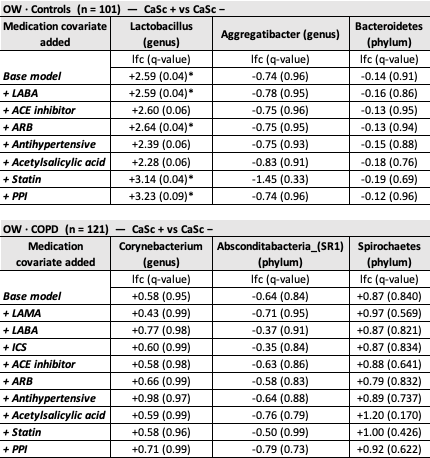


LAMA = long-acting muscarinic antagonist; LABA = long-acting β2-agonist; ICS = inhaled corticosteroid; ACE = angiotensin-converting enzyme; ARB = angiotensin receptor blocker; PPI = proton pump inhibitor.

Taxa shown are those identified as differentially abundant in the primary ANCOMBC2 analysis (Table 3). All models adjusted for age, sex, and smoking status.

Lactobacillus (Table A, green) remained significant after adjustment for LABA (q=0.044), ARB (q=0.041), statin (q=0.004), and PPI (q=0.009), but was borderline non-significant after adjustment for ACE inhibitor (q=0.060), antihypertensive (q=0.056), or aspirin (q=0.062).

**Supplemental S2 Table D. ANCOM-BC2 medication sensitivity analyses: BAL samples – Controls and COPD patients**

Effect of CaSc on differentially abundant taxa (from Table 3) when individual medication classes are added as covariates. Base model: CaSc + age + sex + smoking. Values shown as log-fold change (q-value, BH-corrected). * q < 0.05.


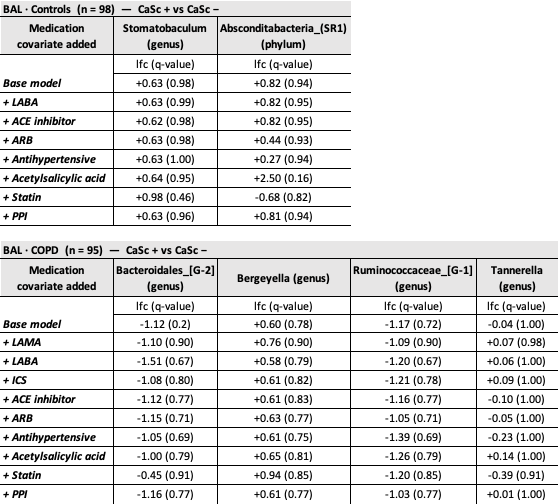


LAMA = long-acting muscarinic antagonist; LABA = long-acting β2-agonist; ICS = inhaled corticosteroid; ACE = angiotensin-converting enzyme; ARB = angiotensin receptor blocker; PPI = proton pump inhibitor.

Taxa shown are those identified as differentially abundant in the primary ANCOMBC2 analysis (Table 3). All models adjusted for age, sex, and smoking status.

Lactobacillus (Table A, green) remained significant after adjustment for LABA (q=0.044), ARB (q=0.041), statin (q=0.004), and PPI (q=0.009), but was borderline non-significant after adjustment for ACE inhibitor (q=0.060), antihypertensive (q=0.056), or aspirin (q=0.062).
