# Supplementary material for: The oral and lower airway microbiota and coronary heart disease in COPD patients and controls
Source: PLoS One. 2026 Jul 16;21(7):e0353738. doi: 10.1371/journal.pone.0353738 (PMC13374919; doi:10.1371/journal.pone.0353738)
Supplement: S3 Table — (DOCX) [file pone.0353738.s007.docx]

**Supplemental S3 Table. Beta diversity (Bray-Curtis, PERMANOVA), marginal R² for CaSc (p-value). Each row shows the effect of calcium score when the row medication is added as a covariate.**

| **Medication added** | **Unadjusted** | **Adjusted†** | **Unadjusted** | **Adjusted†** | **Unadjusted** | **Adjusted†** | **Unadjusted** | **Adjusted†** |
| --- | --- | --- | --- | --- | --- | --- | --- | --- |
|  | **OW · Controls (n = 101)** | | **OW · COPD (n = 121)** | | **BAL · Controls (n = 98)** | | **BAL · COPD (n = 95)** | |
| **None (base model)** | 0.011  (p=0.29) | 0.008  (p=0.67) | 0.008  (p=0.40) | 0.007  (p=0.55) | 0.008  (p=0.82) | 0.007  (p=0.92) | 0.007  (p=0.88) | 0.006  (p=0.96) |
| LAMA | – | – | 0.008  (p=0.41) | 0.008  (p=0.50) | – | – | 0.007  (p=0.85) | 0.006  (p=0.97) |
| LABA | – | – | 0.008  (p=0.46) | 0.007  (p=0.57) | – | – | 0.007  (p=0.87) | 0.006  (p=0.96) |
| Inhaled  corticosteroid | – | – | 0.008  (p=0.49) | 0.007  (p=0.60) | – | – | 0.007  (p=0.86) | 0.006  (p=0.96) |
| ACE inhibitor | 0.011  (p=0.30) | 0.008  (p=0.60) | 0.008  (p=0.38) | 0.008  (p=0.52) | 0.008  (p=0.82) | 0.007  (p=0.93) | 0.008  (p=0.83) | 0.006  (p=0.95) |
| ARB | 0.011  (p=0.32) | 0.008  (p=0.66) | 0.008  (p=0.41) | 0.007  (p=0.59) | 0.008  (p=0.79) | 0.007  (p=0.92) | 0.008  (p=0.86) | 0.007  (p=0.95) |
| Antihypertensive | 0.011  (p=0.25) | 0.008  (p=0.68) | 0.008  (p=0.39) | 0.007  (p=0.59) | 0.008  (p=0.78) | 0.007  (p=0.91) | 0.008  (p=0.83) | 0.007  (p=0.94) |
| Acetylsalicylic acid | 0.014  (p=0.16) | 0.009  (p=0.49) | 0.007  (p=0.58) | 0.007  (p=0.64) | 0.010  (p=0.44) | 0.007  (p=0.82) | 0.007  (p=0.89) | 0.006  (p=0.97) |
| Statin | 0.013  (p=0.20) | 0.010  (p=0.42) | 0.007  (p=0.69) | 0.006  (p=0.80) | 0.011  (p=0.29) | 0.009  (p=0.65) | 0.007  (p=0.90) | 0.006  (p=0.97) |
| PPI | 0.011  (p=0.29) | 0.008  (p=0.50) | 0.008  (p=0.42) | 0.007  (p=0.57) | 0.008  (p=0.81) | 0.007  (p=0.90) | 0.007  (p=0.87) | 0.006  (p=0.97) |

† Adjusted: age, sex, smoking status included as covariates in addition to the listed medication.

– Not applicable: medication class not used in this diagnostic group (e.g., LAMA/LABA/ICS absent in controls).

Base model: CaSc only (unadjusted) or CaSc + age + sex + smoking (adjusted), without any medication covariate.

All p-values are two-sided. None of the CaSc effects reached statistical significance in any model.

OW = oral wash; BAL = bronchoalveolar lavage; LAMA = long-acting muscarinic antagonist; LABA = long-acting β2-agonist; ICS = inhaled corticosteroid; ACE = angiotensin-converting enzyme; ARB = angiotensin receptor blocker; PPI = proton pump inhibitor.
