# Supplementary material for: The oral and lower airway microbiota and coronary heart disease in COPD patients and controls
Source: PLoS One. 2026 Jul 16;21(7):e0353738. doi: 10.1371/journal.pone.0353738 (PMC13374919; doi:10.1371/journal.pone.0353738)
Supplement: S5 Table — (DOCX) [file pone.0353738.s009.docx]

**Supplemental S5 Table. ANCOM-BC2 medication sensitivity analyses: BAL samples in Controls and COPD patients**

Effect of CaSc on differentially abundant taxa (from Table 3) when individual medication classes are added as covariates. Base model: CaSc + age + sex + smoking. Values shown as log-fold change (q-value, BH-corrected). * q < 0.05.

| **BAL · Controls (n = 98) — CaSc+ vs CaSc−** | | |
| --- | --- | --- |
| **Medication covariate added** | **Stomatobaculum**  **(genus)** | **Absconditabacteria_(SR1)**  **(phylum)** |
|  | lfc (q-value) | lfc (q-value) |
| **Base model** | +0.63 (0.98) | +0.82 (0.94) |
| **+ LABA** | +0.63 (0.99) | +0.82 (0.95) |
| **+ ACE inhibitor** | +0.62 (0.98) | +0.82 (0.95) |
| **+ ARB** | +0.63 (0.98) | +0.44 (0.93) |
| **+ Antihypertensive** | +0.63 (1.00) | +0.27 (0.94) |
| **+ Acetylsalicylic acid** | +0.64 (0.95) | +2.50 (0.16) |
| **+ Statin** | +0.98 (0.46) | -0.68 (0.82) |
| **+ PPI** | +0.63 (0.96) | +0.81 (0.94) |

| **BAL · COPD (n = 95) — CaSc+ vs CaSc−** | | | | |
| --- | --- | --- | --- | --- |
| **Medication covariate added** | **Bacteroidales_[G-2]**  **(genus)** | **Bergeyella**  **(genus)** | **Ruminococcaceae_[G-1]**  **(genus)** | **Tannerella**  **(genus)** |
|  | lfc (q-value) | lfc (q-value) | lfc (q-value) | lfc (q-value) |
| **Base model** | -1.12 (0.72) | +0.60 (0.78) | -1.17 (0.72) | -0.04 (1.00) |
| **+ LAMA** | -1.10 (0.90) | +0.76 (0.90) | -1.09 (0.90) | +0.07 (0.98) |
| **+ LABA** | -1.51 (0.67) | +0.58 (0.79) | -1.20 (0.67) | +0.06 (1.00) |
| **+ ICS** | -1.08 (0.80) | +0.61 (0.82) | -1.21 (0.78) | +0.09 (1.00) |
| **+ ACE inhibitor** | -1.12 (0.77) | +0.61 (0.83) | -1.16 (0.77) | -0.10 (1.00) |
| **+ ARB** | -1.15 (0.71) | +0.63 (0.77) | -1.05 (0.71) | -0.05 (1.00) |
| **+ Antihypertensive** | -1.05 (0.69) | +0.61 (0.75) | -1.39 (0.69) | -0.23 (1.00) |
| **+ Acetylsalicylic acid** | -1.00 (0.79) | +0.65 (0.81) | -1.26 (0.79) | +0.14 (1.00) |
| **+ Statin** | -0.45 (0.91) | +0.94 (0.85) | -1.20 (0.85) | -0.39 (0.91) |
| **+ PPI** | -1.16 (0.77) | +0.61 (0.77) | -1.03 (0.77) | +0.01 (1.00) |

LAMA = long-acting muscarinic antagonist; LABA = long-acting β2-agonist; ICS = inhaled corticosteroid; ACE = angiotensin-converting enzyme; ARB = angiotensin receptor blocker; PPI = proton pump inhibitor. Taxa shown are those identified as differentially abundant in the primary ANCOMBC2 analysis (Table 3). All models adjusted for age, sex, and smoking status. No taxa reached statistical significance (q < 0.05) in any model in BAL samples.
