# Supplementary material for: The oral and lower airway microbiota and coronary heart disease in COPD patients and controls
Source: PLoS One. 2026 Jul 16;21(7):e0353738. doi: 10.1371/journal.pone.0353738 (PMC13374919; doi:10.1371/journal.pone.0353738)
Supplement: Attachment — Submitted filename: Response to Editor_ONE-D-26-08565_29may26.docx [file pone.0353738.s012.docx]

Dear Editor,

Thank you for the opportunity to further revise our manuscript and provide a more detailed response to the reviewers.

1. INCLUSION/EXCLUSION CRITERIA (Reviewer 1, Point 1):
Your response clearly and comprehensively explains the study design rationale, including the volunteer-based bronchoscopy approach, the 14-day antibiotic washout period, the safety-driven exclusion criteria for bronchoscopy, and the age requirements. The transparency about the limitations of these design choices (e.g., the potential residual antibiotic effect after 14 days) is appreciated. The addition of expanded text in the Methods section is appropriate.

Remaining request: Please ensure that the updated Methods text in the revised manuscript explicitly lists the primary inclusion and exclusion criteria in a clear, structured manner so that readers can readily assess the study population without needing to refer to the supplementary publications.

We thank the Editor for this request and have further detailed the methods section to include a structured presentation of inclusion and exclusion criteria. The revised text will hopefully allow readers to assess the study population without reference to supplementary publications.

2. MEDICATION CONFOUNDING (Reviewer 1, Point 2):
Your sensitivity analyses (Tables A-D) represent a substantial and commendable analytical addition to this work. The demonstration that the primary finding — the lack of a significant association between airway microbiota and coronary calcium score — remains robust after adjusting for each medication class individually is reassuring and strengthens the manuscript considerably. The observation that Lactobacillus enrichment in oral wash controls with high CaSc remains significant in most medication-adjusted models is also noteworthy.

Remaining request: Please ensure that the new sensitivity analysis tables are included as supplementary material in the revised manuscript, and that the Methods and Discussion sections clearly reference and describe these analyses.

We thank the Editor for this assessment. The sensitivity analysis tables have been included as Supplementary S2-S5 Tables in the revised manuscript. The methods and discussion sections have been updated as follows.

3. ORAL WASH METHODOLOGY (Reviewer 1, Point 3):
Your decision to correct all references from "upper airways" to "oral" microbiome throughout the manuscript is appropriate and appreciated. This clarification meaningfully improves the accuracy of the manuscript.

Remaining request: Please confirm that all instances of "upper airway" have been updated, and that the Discussion acknowledges the scope and limitations of oral wash as a proxy for microbiome assessment in this context.

We confirm that all instances of “upper airway microbiome” have been replaced with “oral microbiome” throughout the manuscript and added it as a limitation.

4. DISCUSSION OF BACTERIAL COMMUNITIES (Reviewer 1, Point 4 - Discussion):
Thank you for expanding the Discussion section to better address the roles of specific bacterial taxa identified. This is an important improvement that strengthens the scientific rigor of the article.

Remaining request: Please ensure that the expanded discussion specifically addresses the biologically plausible mechanisms linking the oral microbiome (particularly Lactobacillus enrichment in high CaSc controls) with cardiovascular health, as this is a key finding of the study.

We thank the Editor for this request. The discussion section has been expanded to address the plausible mechanisms linking the oral microbiome to coronary heart disease.

5. PICRUST2 FUNCTIONAL ANALYSIS (Reviewer 1, Point 4 - Bioinformatics):
I commend you for conducting the PICRUSt2 analysis in response to the reviewer's suggestion. Your finding that no significant functional pathway differences were detected between high and low calcium score groups is an important negative result. Your explanation of the inherent limitations of 16S-based functional inference at genus level (e.g., the Lactobacillus/Clostridium and Corynebacterium/Mycobacterium examples) is scientifically sound and provides appropriate caution.

The decision not to include PICRUSt2 results in the main manuscript is acceptable given the null findings and methodological limitations. However, please include the PICRUSt2 results as a supplementary table, as this adds transparency and will be valuable to readers interested in the functional microbiome aspects.

We thank the editor for this request. PICRUSt2 results are now included in the discussion and added as Supplementary S3-S4 Figure.
