# Supplementary material for: The oral and lower airway microbiota and coronary heart disease in COPD patients and controls
Source: PLoS One. 2026 Jul 16;21(7):e0353738. doi: 10.1371/journal.pone.0353738 (PMC13374919; doi:10.1371/journal.pone.0353738)
Supplement: Attachment — Submitted filename: Response to referee PLOS One May 2026.docx [file pone.0353738.s011.docx]

Dear Editor,

We thank you for the opportunity to revise our manuscript and provide a point-by-point response to the reviewers. We have tried to improve the manuscript, particularly considering the advice of the editor to clarify the study design and methods, ensure that the statistical approach and interpretation are fully justified, and align the conclusions closely with the data presented.

Reviewer #1: Review report April 17, 2026
The submitted manuscript entitled “The upper and lower airway microbiota and coronary heart disease in COPD patients
and controls” has discussed timely interesting topic, the connection between the respiratory microbiota and CHD patients. However, some weak points listed below make the publication of this article in its current status incompetent.

1- Microbiome is diversified subject and the exclusion and inclusion criteria considered in this study design are not satisfying.

We admit being a bit confused about this statement; that is, it is not inherently clear to us what is meant by "Microbiome is diversified subject". We have interpreted this statement to mean that the microbiome as a study term is a diverse topic, in that it encompasses many potential connections, ecological niches or habitats which may or may not impact health. This we certainly agree with. However, to link that with which inclusion or exclusion criteria are satisfying or not in a study on the potential association between the airways microbiome and cardiac health is not immediately obvious to us.

Our study is observational, and therefore by default vulnerable to selection bias. However, studying the lower airways microbiome by bronchoscopic sampling includes having a procedure performed which may mean some discomfort to the study subjects. In our cohort, all procedures were performed on volunteers, with no financial gain, or any other gain, as the bronchoscopies were not performed for any diagnostic purposes. This design avoids the bias of including patients being examined for a reason, thus being likely to have a disease characteristic which may impact the results, like an ongoing COPD exacerbation, pneumonia, or potentially lung cancer.

Now, although bronchoscopy is overwhelmingly a safe procedure, it is an invasive procedure with the potential for some side effects, notably A) bleeding (not from washings, but in some patients, we included bronchial brushes and bronchial biopsies. Also, we needed to be able to sample unexpected visual endobronchial changes, thus bleeding risk was always considered), B) inducing bronchospasm, C) allergy to procedural sedation, D) cardiac events like atrial fibrillation, or acute coronary events, and E) adverse reactions to alfentanil (respiratory depression). These effects are all rare, and mostly manageable in the bronchoscopy theatre, but of course, we took every precaution possible to avoid them. The meant that having a history of unstable coronary disease, atrial fibrillation, recent thromboembolic disease, use of anti-coagulants other than acetyl salicylic acid, and known allergy to any of the procedural medications were contraindications to participate.

To avoid bronchospasm which could happen in COPD (or asthma) patients; both patients and controls received a dose of salbutamol before the bronchoscopy (standard during the lung function test taken before the bronchoscopy), and they were only examined while in the stable state. Further, we avoided taking bronchoalveolar lavage if the lung function corresponded to an FEV_1_ less than 30% or 1 liter. As part of the MicroCOPD study we followed our patients closely for any discomfort, complications or side effects, and this has been published^1^.

Now, one could argue that this excludes unstable and potentially more severely heart disease afflicted COPD patients and controls, but such were the restraints from any ethical perspective. Also, the exclusion criteria above applied equally to all study participants. In addition to this, all adults in the study were > 40 years of age, a standard requirement for diagnosing COPD, which almost never starts before the age of 40 (in such cases it would usually be confounding asthma).

Another requirement was not having taken any antibiotic within the last 14 days (for any indication). Obviously, this was chosen to avoid impacting the microbiome. In retrospect, 14 days were perhaps a bit short, as some residual effect of antibiotics could be present after 15 days. We chose 14 days to avoid excluding an important group of COPD patients, namely those who frequently exacerbate, and therefore are more likely to have several courses of antibiotics taken during a year. However, in previous publications from the MicroCOPD study, other reviewers have pointed to these 14 days as perhaps being a source of impact on microbial profiling, and we therefore performed a journal review and analyzed use of antibiotics the last month, 3 months, and 12 months. This has been published before^2,3^. Briefly, 3% (6 subjects) had taken AB < 4 weeks before the bronchoscopy and 11% < 3 months before.

We did not have an upper age limit in the study and included both sexes. We allowed smokers and non-smokers (a smoking history of > 10 pack years was necessary in COPD, a standard requirement in most studies to avoid confounding with asthma), and recorded all medication use participants had, which we address in detail at point 2.

We take the reviewers' and editors' comments to mean that we should provide more details on inclusion and exclusion criteria in the current paper for increased clarity for the reader, and we have added some text to the methods section to address this.

2- COPD or CHD patients certainly receive non-antibiotic medicines which will directly or indirectly change host-microbiome interactions. Do all participants have same medication regimen?

As our controls were recruited from previous population cohorts, and COPD patients have many comorbidities, our study participants did indeed use a large range of non-antibiotic medications as the reviewer states.

Now, non-antibiotic medications may mean less than the reviewer believes. One medication we have been concerned about in particular, is inhaled corticosteroids (ICS), which is taken by several COPD patients as part of their medication regimen but is not taken by lung healthy controls. Corticosteroids are immunosuppressants, and oral thrash (mild local fungal disease usually by candida) is a common side effect. Thus, it would be logical that inhaled corticosteroids could impact both the oral and lower airways bacterial microbiome. Now, the study question in the current study was whether the oral or lower airways microbiome was associated with coronary heart disease. This we examined both in controls and COPD patients separately, and therefore in controls ICS would regardless not be a factor.

The effects of ICS in COPD we have examined in some detail in our previous studies. The effects on the fungal microbiome has been there^4^, but on the bacterial microbiome no difference in either diversity or taxonomy (differential abundance, DA) was seen between patients who used and those who did not use ICS^3^.

We performed both unadjusted and adjusted analyses on our study question, with reasonably similar results. We are pleased to know we have performed the largest single center study with bronchoscopy sampling of healthy adults to date, yet our sample size is still vulnerable to lack of strength if we include too many covariables. Knowing ICS did not impact the microbiome in our cohort we chose to exclude them here.

Perhaps more difficult is handling medications for heart disease. Obviously, many of our patients had known heart disease, and took medications as such. Of particular difficulty would be handling of statins since statin use could in theory elevate or mask significant calcium scores. However, the distribution of medication use in our study sample is actually reasonably even, considering the COPD patients in general are prone to have more comorbidities:

|  | Controls (n=101) | | COPD patients (n=127) | |  |
| --- | --- | --- | --- | --- | --- |
| *Medication use* | n | % | n | % | p |
| Long acting muscarinic antagonists (LAMA) | 0 | 0 | 79 | 62.2 | <0.01 |
| Long acting beta2 agonists | 0 | 0 | 87 | 68.5 | <0.01 |
| Inhaled corticosteroids | 0 | 0 | 80 | 63.0 | <0.01 |
| Angiotensin Converting Enzyme Inhibitors | 2 | 2.0 | 3 | 2.4 | 0.85 |
| Angiotensin Receptor Blockers | 10 | 9.9 | 17 | 13.4 | 0.42 |
| Any anti-hypertensive drug | 18 | 17.8 | 29 | 22.8 | 0.35 |
| Acetyl salicylic acid | 13 | 12.9 | 34 | 26.7 | 0.01 |
| Statin | 21 | 20.8 | 37 | 29.1 | 0.15 |
| Proton pump inhibitors | 6 | 5.9 | 19 | 15.0 | 0.03 |

The most important questions are perhaps whether there are systematic differences between those with and without CHD in either controls or COPD patients:

| In Controls only: |  |  |  |  |  |
| --- | --- | --- | --- | --- | --- |
|  | no sign CaSc | | Sign CaSc | |  |
| *Medication use* | n | % | n | % | p |
| Angiotensin Receptor Blockers | 7 | 10.0 | 3 | 9.7 | 0.96 |
| Any anti-hypertensive drug | 11 | 15.7 | 7 | 22.6 | 0.41 |
| Acetyl salicylic acid | 5 | 7.1 | 8 | 25.8 | 0.01 |
| Statin | 6 | 8.6 | 15 | 48.4 | <0.01 |
| Proton pump inhibitors | 4 | 5.7 | 2 | 6.5 | 0.60 |
|  |  |  |  |  |  |
|  |  |  |  |  |  |
| In COPD only: |  |  |  |  |  |
|  | no sign CaSc | | Sign CaSc | |  |
| *Medication use* | n | % | n | % | p |
| Long acting muscarinic antagonists (LAMA) | 30 | 51.7 | 48 | 71.6 | 0.02 |
| Long acting beta2 agonists | 37 | 63.8 | 49 | 73.1 | 0.26 |
| Inhaled corticosteroids | 34 | 58.6 | 45 | 67.2 | 0.32 |
| Angiotensin Converting Enzyme Inhibitors | 1 | 1.7 | 2 | 3.0 | 0.55 |
| Angiotensin Receptor Blockers | 6 | 10.3 | 9 | 13.4 | 0.60 |
| Any anti-hypertensive drug | 9 | 15.5 | 18 | 26.9 | 0.12 |
| Acetyl salicylic acid | 8 | 13.8 | 24 | 35.8 | 0.01 |
| Statin | 8 | 13.8 | 27 | 40.3 | <0.01 |
| Proton pump inhibitors | 6 | 10.3 | 13 | 19.4 | 0.16 |

Unsurprisingly, having a high Calcium Score was consistent with higher use of statins and platelet inhibitors, as many subjects knew from before they had CHD.

Now, we have not come across a great deal of evidence that the oral or the lower airways microbiome is impacted by these medications.

In a Japanese study on 82 older subjects with "with a heterogeneous background of comorbidities". In that study, there was some evidence that salivary streptococcal abundance was a bit lower in subjects taking statins.

However, in a Canadian study published in PLoS One 2021 Dec 9;16(12):e0261032. doi: [10.1371/journal.pone.0261032](https://doi.org/10.1371/journal.pone.0261032)), more than 1000 subjects had saliva samples profiled, and correlated with medication use. Although a few statistically DA taxa were found, the conclusion of the authors were:

*"The results from this study show negligible effect of commonly used medications on microbial diversity and small differences in the relative abundance of specific taxa, suggesting a minimal influence of commonly used medication on the salivary microbiome of individuals living without major chronic conditions."*

On the lower airways microbiome, we have found no previously published data.

As expected in a clinical cohort of COPD patients, participants used a range of medications including inhaled bronchodilators (LAMA/LABA), inhaled corticosteroids, statins, ACE inhibitors, ARBs, acetylsalicylic acid, and proton pump inhibitors. We have re-run all multivariable diversity and DA analyses, adding each (one by one to avoid collinearity and overstretching our regression models) of the medications listed in the tables above.

For alpha diversity (Shannon Index), the CaSc>100 effect estimate remained stable across all medication-adjusted models, with no medication changing the non-significant association (Table A):

**Supplementary Table: Medication sensitivity analyses — effect of CaSc on alpha and beta diversity**

**Table A. Alpha diversity (Shannon index) — linear regression coefficient (β) for sign_CACS (p-value). Each row shows the effect of calcium score in alpha diversity when the row medication is added as a covariate.**

| **Alpha diversity (Shannon index) — β for CaSc (p-value)** | | | | | | | | |
| --- | --- | --- | --- | --- | --- | --- | --- | --- |
|  | **Unadjusted** | **Adjusted†** | **Unadjusted** | **Adjusted†** | **Unadjusted** | **Adjusted†** | **Unadjusted** | **Adjusted†** |
| **Medication added** | **OW · Controls (n = 101)** | | **OW · COPD (n = 121)** | | **BAL · Controls (n = 98)** | | **BAL · COPD (n = 95)** | |
| **None (base model)** | **0.127 (p=0.121)** | **0.142 (p=0.136)** | **-0.109 (p=0.239)** | **-0.010 (p=0.922)** | **0.076 (p=0.658)** | **0.061 (p=0.758)** | **-0.144 (p=0.404)** | **-0.256 (p=0.176)** |
| LAMA | – | – | -0.093 (p=0.321) | 0.010 (p=0.921) | – | – | -0.153 (p=0.383) | -0.274 (p=0.158) |
| LABA | – | – | -0.101 (p=0.275) | -0.007 (p=0.946) | – | – | -0.144 (p=0.408) | -0.257 (p=0.177) |
| Inhaled corticosteroid | – | – | -0.102 (p=0.272) | -0.007 (p=0.943) | – | – | -0.138 (p=0.425) | -0.255 (p=0.181) |
| ACE inhibitor | 0.119 (p=0.147) | 0.135 (p=0.156) | -0.104 (p=0.267) | -0.008 (p=0.941) | 0.071 (p=0.680) | 0.057 (p=0.775) | -0.138 (p=0.424) | -0.262 (p=0.168) |
| ARB | 0.126 (p=0.118) | 0.140 (p=0.135) | -0.112 (p=0.226) | -0.015 (p=0.886) | 0.078 (p=0.647) | 0.062 (p=0.752) | -0.147 (p=0.396) | -0.269 (p=0.158) |
| Antihypertensive | 0.140 (p=0.085) | 0.147 (p=0.117) | -0.135 (p=0.144) | -0.037 (p=0.716) | 0.093 (p=0.588) | 0.071 (p=0.719) | -0.153 (p=0.381) | -0.270 (p=0.158) |
| Acetylsalicylic acid | 0.143 (p=0.092) | 0.154 (p=0.114) | -0.090 (p=0.352) | 0.008 (p=0.939) | 0.114 (p=0.525) | 0.086 (p=0.672) | -0.157 (p=0.386) | -0.250 (p=0.197) |
| Statin | 0.167 (p=0.070) | 0.178 (p=0.084) | -0.134 (p=0.168) | -0.036 (p=0.737) | 0.110 (p=0.569) | 0.111 (p=0.605) | -0.133 (p=0.468) | -0.242 (p=0.218) |
| PPI | 0.128 (p=0.120) | 0.141 (p=0.140) | -0.082 (p=0.364) | 0.015 (p=0.883) | 0.079 (p=0.642) | 0.054 (p=0.783) | -0.125 (p=0.474) | -0.239 (p=0.211) |

Beta diversity (Bray-Curtis PERMANOVA) showed no significant difference in community-level composition and with consistently small R2-values across all strata and all medication-adjusted models, indicating that community composition does not differ by CaSc status regardless of medication adjustment (Table B).

**Table B. Beta diversity (Bray-Curtis, PERMANOVA) — marginal R² for CaSc (p-value). Each row shows the effect of calcium score when the row medication is added as a covariate.**

| **Beta diversity (Bray-Curtis PERMANOVA) — R² for CaSc (p-value)** | | | | | | | | |
| --- | --- | --- | --- | --- | --- | --- | --- | --- |
|  | **Unadjusted** | **Adjusted†** | **Unadjusted** | **Adjusted†** | **Unadjusted** | **Adjusted†** | **Unadjusted** | **Adjusted†** |
| **Medication added** | **OW · Controls (n = 101)** | | **OW · COPD (n = 121)** | | **BAL · Controls (n = 98)** | | **BAL · COPD (n = 95)** | |
| **None (base model)** | **0.011 (p=0.286)** | **0.008 (p=0.671)** | **0.008 (p=0.403)** | **0.007 (p=0.547)** | **0.008 (p=0.820)** | **0.007 (p=0.917)** | **0.007 (p=0.876)** | **0.006 (p=0.962)** |
| LAMA | – | – | 0.008 (p=0.414) | 0.008 (p=0.499) | – | – | 0.007 (p=0.848) | 0.006 (p=0.965) |
| LABA | – | – | 0.008 (p=0.463) | 0.007 (p=0.567) | – | – | 0.007 (p=0.872) | 0.006 (p=0.962) |
| Inhaled corticosteroid | – | – | 0.008 (p=0.487) | 0.007 (p=0.603) | – | – | 0.007 (p=0.864) | 0.006 (p=0.955) |
| ACE inhibitor | 0.011 (p=0.299) | 0.008 (p=0.599) | 0.008 (p=0.381) | 0.008 (p=0.522) | 0.008 (p=0.822) | 0.007 (p=0.927) | 0.008 (p=0.834) | 0.006 (p=0.954) |
| ARB | 0.011 (p=0.321) | 0.008 (p=0.664) | 0.008 (p=0.412) | 0.007 (p=0.589) | 0.008 (p=0.792) | 0.007 (p=0.917) | 0.008 (p=0.855) | 0.007 (p=0.945) |
| Antihypertensive | 0.011 (p=0.254) | 0.008 (p=0.677) | 0.008 (p=0.392) | 0.007 (p=0.593) | 0.008 (p=0.783) | 0.007 (p=0.912) | 0.008 (p=0.833) | 0.007 (p=0.938) |
| Acetylsalicylic acid | 0.014 (p=0.160) | 0.009 (p=0.490) | 0.007 (p=0.577) | 0.007 (p=0.637) | 0.010 (p=0.440) | 0.007 (p=0.821) | 0.007 (p=0.889) | 0.006 (p=0.965) |
| Statin | 0.013 (p=0.200) | 0.010 (p=0.416) | 0.007 (p=0.689) | 0.006 (p=0.804) | 0.011 (p=0.290) | 0.009 (p=0.648) | 0.007 (p=0.902) | 0.006 (p=0.967) |
| PPI | 0.011 (p=0.293) | 0.008 (p=0.647) | 0.008 (p=0.416) | 0.007 (p=0.568) | 0.008 (p=0.807) | 0.007 (p=0.904) | 0.007 (p=0.871) | 0.006 (p=0.972) |

† Adjusted: age, sex, smoking status included as covariates in addition to the listed medication.

– Not applicable: medication class not used in this diagnostic group (e.g., LAMA/LABA/ICS absent in controls).

Base model (yellow): sign_CACS only (unadjusted) or sign_CACS + age + sex + smoking (adjusted), without any medication covariate.

All p-values are two-sided. None of the sign_CACS effects reached statistical significance in any model.

OW = oral wash; BAL = bronchoalveolar lavage; LAMA = long-acting muscarinic antagonist; LABA = long-acting β2-agonist; ICS = inhaled corticosteroid; ACE = angiotensin-converting enzyme; ARB = angiotensin receptor blocker; PPI = proton pump inhibitor.

Regarding differential abundance, we conducted similar sensitivity analyses. The base model represents the adjusted estimate (CaSc + age + sex + smoking) and therefore does not directly replicate the unadjusted column of Table 3 in the manuscript. Taxa previously significant only in unadjusted models were not significant in the adjusted base model, consistent with confounding by age, sex, or smoking. Lactobacillus remained significantly enriched in OW samples from controls in high CaSc-participants after adjustment for LABA (q=0.044), ARB (q=0.041), statins (q=0.004), and PPI (q=0.009). No other taxa showed significant differential abundance in any medication-adjusted model across the remaining subgroups.

To sum up, we could find no significant effect from use of medications on the association between the microbiome and the presence of CHD. We realize from the reviewer's comment that this needs to be mentioned in the manuscript and have therefore expanded the methods section and the discussion a bit to address the potential relationship between medication use and the microbiome.

**Supplementary Table: ANCOM-BC2 medication sensitivity analyses**

Effect of CaSc on differentially abundant taxa (from Table 3) when individual medication classes are added as covariates. Base model: CaSc + age + sex + smoking. Values shown as log-fold change (q-value, BH-corrected). * q < 0.05. Green shading = significant finding.

**Table C. OW samples — Controls**

| **OW · Controls (n = 101) — CaSc + vs CaSc −** | | | |
| --- | --- | --- | --- |
| **Medication covariate added** | **Lactobacillus (genus)** | **Aggregatibacter (genus)** | **Bacteroidetes (phylum)** |
|  | lfc (q-value) | lfc (q-value) | lfc (q-value) |
| **Base model** | **+2.59 (0.035)*** | -0.74 (0.959) | -0.14 (0.911) |
| + LABA | **+2.59 (0.044)*** | -0.78 (0.954) | -0.16 (0.859) |
| + ACE inhibitor | +2.60 (0.060) | -0.75 (0.964) | -0.13 (0.945) |
| + ARB | **+2.64 (0.041)*** | -0.75 (0.953) | -0.13 (0.942) |
| + Antihypertensive | +2.39 (0.056) | -0.75 (0.927) | -0.15 (0.878) |
| + Acetylsalicylic acid | +2.28 (0.062) | -0.83 (0.907) | -0.18 (0.759) |
| + Statin | **+3.14 (0.004)*** | -1.45 (0.331) | -0.19 (0.690) |
| + PPI | **+3.23 (0.009)*** | -0.74 (0.964) | -0.12 (0.957) |

**Table D. OW samples — COPD patients**

| **OW · COPD (n = 121) — CaSc + vs CaSc −** | | | |
| --- | --- | --- | --- |
| **Medication covariate added** | **Corynebacterium (genus)** | **Absconditabacteria_(SR1) (phylum)** | **Spirochaetes (phylum)** |
|  | lfc (q-value) | lfc (q-value) | lfc (q-value) |
| **Base model** | +0.58 (0.994) | -0.64 (0.840) | +0.87 (0.840) |
| + LAMA | +0.43 (0.997) | -0.71 (0.949) | +0.97 (0.569) |
| + LABA | +0.77 (0.975) | -0.37 (0.913) | +0.87 (0.821) |
| + ICS | +0.60 (0.985) | -0.35 (0.842) | +0.87 (0.834) |
| + ACE inhibitor | +0.58 (0.980) | -0.63 (0.859) | +0.88 (0.641) |
| + ARB | +0.66 (0.994) | -0.58 (0.832) | +0.79 (0.832) |
| + Antihypertensive | +0.98 (0.969) | -0.64 (0.881) | +0.89 (0.737) |
| + Acetylsalicylic acid | +0.59 (0.997) | -0.76 (0.791) | +1.20 (0.170) |
| + Statin | +0.58 (0.956) | -0.50 (0.999) | +1.00 (0.426) |
| + PPI | +0.71 (0.986) | -0.79 (0.725) | +0.92 (0.622) |

**Table C. BAL samples — Controls**

| **BAL · Controls (n = 98) — CaSc + vs CaSc −** | | |
| --- | --- | --- |
| **Medication covariate added** | **Stomatobaculum (genus)** | **Absconditabacteria_(SR1) (phylum)** |
|  | lfc (q-value) | lfc (q-value) |
| **Base model** | +0.63 (0.977) | +0.82 (0.941) |
| + LABA | +0.63 (0.987) | +0.82 (0.948) |
| + ACE inhibitor | +0.62 (0.979) | +0.82 (0.947) |
| + ARB | +0.63 (0.978) | +0.44 (0.929) |
| + Antihypertensive | +0.63 (1.000) | +0.27 (0.935) |
| + Acetylsalicylic acid | +0.64 (0.946) | +2.50 (0.159) |
| + Statin | +0.98 (0.463) | -0.68 (0.818) |
| + PPI | +0.63 (0.960) | +0.81 (0.944) |

**Table D. BAL samples — COPD patients**

| **BAL · COPD (n = 95) — CaSc + vs CaSc −** | | | | |
| --- | --- | --- | --- | --- |
| **Medication covariate added** | **Bacteroidales_[G-2] (genus)** | **Bergeyella (genus)** | **Ruminococcaceae_[G-1] (genus)** | **Tannerella (genus)** |
|  | lfc (q-value) | lfc (q-value) | lfc (q-value) | lfc (q-value) |
| **Base model** | -1.12 (0.715) | +0.60 (0.795) | -1.17 (0.715) | -0.04 (1.000) |
| + LAMA | -1.10 (0.899) | +0.76 (0.899) | -1.09 (0.899) | +0.07 (0.979) |
| + LABA | -1.51 (0.674) | +0.58 (0.789) | -1.20 (0.674) | +0.06 (1.000) |
| + ICS | -1.08 (0.779) | +0.61 (0.820) | -1.21 (0.779) | +0.09 (1.000) |
| + ACE inhibitor | -1.12 (0.772) | +0.61 (0.828) | -1.16 (0.772) | -0.10 (1.000) |
| + ARB | -1.15 (0.705) | +0.63 (0.767) | -1.05 (0.705) | -0.05 (1.000) |
| + Antihypertensive | -1.05 (0.689) | +0.61 (0.752) | -1.39 (0.689) | -0.23 (1.000) |
| + Acetylsalicylic acid | -1.00 (0.794) | +0.65 (0.811) | -1.26 (0.794) | +0.14 (1.000) |
| + Statin | -0.45 (0.910) | +0.94 (0.853) | -1.20 (0.853) | -0.39 (0.910) |
| + PPI | -1.16 (0.769) | +0.61 (0.769) | -1.03 (0.769) | +0.01 (1.000) |

Yellow rows = base model (no medication covariate). Blue headers = controls; purple headers = COPD patients.

LAMA = long-acting muscarinic antagonist; LABA = long-acting β2-agonist; ICS = inhaled corticosteroid; ACE = angiotensin-converting enzyme; ARB = angiotensin receptor blocker; PPI = proton pump inhibitor.

Taxa shown are those identified as differentially abundant in the primary ANCOMBC2 analysis (Table 3). All models adjusted for age, sex, and smoking status.

Lactobacillus (Table A, green) remained significant after adjustment for LABA (q=0.044), ARB (q=0.041), statin (q=0.004), and PPI (q=0.009), but was borderline non-significant after adjustment for ACE inhibitor (q=0.060), antihypertensive (q=0.056), or aspirin (q=0.062).

3- Oral wash does not reflect the upper respiratory tract. It is rather reflective of oral microbiome, which is directly influenced by food and drink styles, general gum health or too many other factors.

We agree to a point. It is well known the nasal and oral microbiome differs but both compartments belong to the upper airways. Oral wash does not reflect the nasal microbiome and is indeed impacted by dental health, and possibly alcohol use and food habits (although the evidence for the latter is actually still surprisingly thin per our reading of the medical literature). However, the fact that dental health has been correlated with several major health issues including heart disease is indeed also one of our major reasons for wanting to study it.

We interpret the reviewer to mean that we may have confused the descriptions in the text to imply we thought of the oral microbiome as equal to the (full) upper respiratory microbiome. We apologize for any such confusion and have changed all use of upper airways to oral in the text to clarify.

4- Ignoring the roles of different bacterial communities in the discussion chapter weakens the scientific approach of this article. Furthermore, bioinformatics could have been better if the PICRUST analysis was added.

We agree with the referee that the functional role of the microbiome is a very important issue, which expands on examining the composition of taxa. Functionality is more difficult to address and is usually examined by whole genome sequencing. However, whole genome sequencing is challenging in low biomass samples. PICRUST analysis is an indirect measure of functionality when using 16S data to infer functionality based on known associations of common metabolic pathways. Due to the reviewer's suggestions, we took a look at PICRUST for our study cohort:

**Table G. PICRUSt2 functional pathway prediction on OW and BAL samples stratified for low or high CaSc.**

**Table H. PICRUSt2 functional pathway prediction on OW and BAL samples stratified for low or high CaSc in COPD patients and healthy controls.**

The PICRUSt2 functional pathway analysis revealed no statistically significant differences in predicted metabolic pathway abundances between CaSc >100 and CaSc < 100 participants after correction for multiple comparisons in either OW or BAL. This was true for the whole group (Table G) and controls and COPD separately (Table H).

PICRUSt2 functional predictions are based on reference genomes annotated at species level. As 16S rRNA gene sequencing in this study provided genus-level resolution at best, there is inherent uncertainty in mapping predicted pathway abundances to observed taxa. Functional predictions should therefore be interpreted with particular caution, as metabolic capacity can vary substantially between species within the same genus. For example, GABA degradation V (PWY-5022) is annotated in *Clostridium aminobutyricum*, whereas the found taxon in our dataset was *Lactobacillus*, a related but distinct *Bacillota* genus. Similarly, mycothiol biosynthesis is documented in *Mycobacterium* and *Streptomyces*, whereas the taxon in our dataset was *Corynebacterium*. This species to genus uncertainty illustrates the inherent limitations of 16S-based functional inference and shotgun metagenomics would be required to potentially draw clearer conclusions about functional pathways in these communities.

Given these caveats, and that the paper is already full of results, we prefer not to add the PICRUSt2 analyses. We thank the reviewer for pointing out the importance of functionality though and intend to examine this in more detail in our MicroCOPD study at a later time.

1.	Leiten, E. O. *et al.* Complications and discomfort after research bronchoscopy in the MicroCOPD study. *BMJ Open Respir Res* **7**, (2020).

2.	Kringeland, G. D. *et al.* Antimicrobial resistance genes and antibiotic use in chronic lung disease: a bronchoscopy study of the lower airways microbiome. *BMJ Open Respir Res* **13**, e003864 (2026).

3.	Tangedal, S. *et al.* Lower airway microbiota in COPD and healthy controls. *Thorax* thorax–2023 (2024).

4.	Martinsen, E. M. H. *et al.* The pulmonary mycobiome-A study of subjects with and without chronic obstructive pulmonary disease. *PLoS One* **16**, e0248967 (2021).

Reviewer #2: Possible association between airway microbiome and coronary atherosclerosis is an emerging research field and i see this study as a great effort to find any association. it is technically sound and presented in easy to understand fashion.

We thank the reviewer for those kind words. We have worked quite some time on presentation of our study results and are grateful for the appreciation of our efforts.
