# Supplementary material for: The oral and lower airway microbiota and coronary heart disease in COPD patients and controls
Source: PLoS One. 2026 Jul 16;21(7):e0353738. doi: 10.1371/journal.pone.0353738 (PMC13374919; doi:10.1371/journal.pone.0353738)
Supplement: Peer review history

# Peer review: Decision Letter 0

**DOI:** 10.1371/journal.pone.0353738.r001

**Authors:** Jamal Akhtar

26 Apr 2026

PONE-D-26-08565

The upper and lower airway microbiota and coronary heart disease in COPD patients and controls

PLOS One

Dear Dr. Svendsen,

Thank you for submitting your manuscript to PLOS ONE. After careful consideration, we feel that it has merit but does not fully meet PLOS ONE’s publication criteria as it currently stands. Therefore, we invite you to submit a revised version of the manuscript that addresses the points raised during the review process.

Thank you for submitting your manuscript, “The upper and lower airway microbiota and coronary heart disease in COPD patients and controls,” to PLOS One. Following editorial assessment and external peer review, the manuscript received divergent recommendations, with Reviewer 1 recommending rejection and Reviewer 2 recommending acceptance. Based on these mixed reviews, the decision is Major Revision.

Please revise the manuscript carefully and provide a detailed, point-by-point response to all reviewer comments. The revision should address the major concerns raised by Reviewer 1, clarify the study design and methods, ensure that the statistical approach and interpretation are fully justified, and align the conclusions closely with the data presented. Please submit both a clean revised manuscript and a marked version showing all changes.

Please note that resubmission does not guarantee acceptance, and the revised manuscript may be returned for further peer review before a final decision is made.

Please submit your revised manuscript by Jun 10 2026 11:59PM. If you will need more time than this to complete your revisions, please reply to this message or contact the journal office at plosone@plos.org. When you're ready to submit your revision, log on to https://www.editorialmanager.com/pone/ and select the 'Submissions Needing Revision' folder to locate your manuscript file.

Please include the following items when submitting your revised manuscript:

- A letter that responds to each point raised by the academic editor and reviewer(s). You should upload this letter as a separate file labeled 'Response to Reviewers'.
- A marked-up copy of your manuscript that highlights changes made to the original version. You should upload this as a separate file labeled 'Revised Manuscript with Track Changes'.
- An unmarked version of your revised paper without tracked changes. You should upload this as a separate file labeled 'Manuscript'.

If you would like to make changes to your financial disclosure, please include your updated statement in your cover letter. Guidelines for resubmitting your figure files are available below the reviewer comments at the end of this letter.

If applicable, we recommend that you deposit your laboratory protocols in protocols.io to enhance the reproducibility of your results. Protocols.io assigns your protocol its own identifier (DOI) so that it can be cited independently in the future. For instructions see: https://journals.plos.org/plosone/s/submission-guidelines#loc-laboratory-protocols. Additionally, PLOS ONE offers an option for publishing peer-reviewed Lab Protocol articles, which describe protocols hosted on protocols.io. Read more information on sharing protocols at https://plos.org/protocols?utm_medium=editorial-email&utm_source=authorletters&utm_campaign=protocols.

As the corresponding author, your ORCID iD is verified in the submission system and will appear in the published article. PLOS supports the use of ORCID, and we encourage all coauthors to register for an ORCID iD and use it as well. Please encourage your coauthors to verify their ORCID iD within the submission system before final acceptance, as unverified ORCID iDs will not appear in the published article. *Only*  the individual author can complete the verification step; PLOS staff *cannot*  verify ORCID iDs on behalf of authors.

We look forward to receiving your revised manuscript.

Kind regards,

Jamal Akhtar

Academic Editor

PLOS One

Journal Requirements:

If the reviewer comments include a recommendation to cite specific previously published works, please review and evaluate these publications to determine whether they are relevant and should be cited. There is no requirement to cite these works unless the editor has indicated otherwise.

Additional Editor Comments:

Dear Dr. Svendsen,

Thank you for submitting your manuscript, “The upper and lower airway microbiota and coronary heart disease in COPD patients and controls,” to PLOS One. Following editorial assessment and external peer review, the manuscript received divergent recommendations, with Reviewer 1 recommending rejection and Reviewer 2 recommending acceptance. Based on these mixed reviews, the decision is Major Revision.

Please revise the manuscript carefully and provide a detailed, point-by-point response to all reviewer comments. The revision should address the major concerns raised by Reviewer 1, clarify the study design and methods, ensure that the statistical approach and interpretation are fully justified, and align the conclusions closely with the data presented. Please submit both a clean revised manuscript and a marked version showing all changes.

Please note that resubmission does not guarantee acceptance, and the revised manuscript may be returned for further peer review before a final decision is made.

Sincerely,

Jamal Akhtar

Academic Editor, PLOS One

Journal requirements:

When submitting your revision, we need you to address these additional requirements.

1. Please ensure that your manuscript meets PLOS ONE's style requirements, including those for file naming. The PLOS ONE style templates can be found at

https://journals.plos.org/plosone/s/file?id=wjVg/PLOSOne_formatting_sample_main_body.pdf and

https://journals.plos.org/plosone/s/file?id=ba62/PLOSOne_formatting_sample_title_authors_affiliations.pdf

2. We note that the grant information you provided in the ‘Funding Information’ and ‘Financial Disclosure’ sections do not match.

When you resubmit, please ensure that you provide the correct grant numbers for the awards you received for your study in the ‘Funding Information’ section.

3. Thank you for stating the following in the Competing Interests section: [I have read the journal's policy and the authors of this manuscript have the following competing interests: Christina Svendsen declares lecture fees from AstraZeneca and Boehringer Ingelheim. Rune Nielsen declares grants from Sanofi and AstraZeneca. Tomas Eagan declares research grant from GSK and lecture fees from Boehringer Ingelheim and AstraZeneca.  This does not alter our adherence to PLOS ONE policies on sharing data and materials.].

Please confirm that this does not alter your adherence to all PLOS ONE policies on sharing data and materials, by including the following statement: ""This does not alter our adherence to  PLOS ONE policies on sharing data and materials.” (as detailed online in our guide for authors http://journals.plos.org/plosone/s/competing-interests).  If there are restrictions on sharing of data and/or materials, please state these. Please note that we cannot proceed with consideration of your article until this information has been declared.

4. Please include your updated Competing Interests statement in your cover letter; we will change the online submission form on your behalf.

[Note: HTML markup is below. Please do not edit.]

Reviewers' comments:

Reviewer's Responses to Questions

**Comments to the Author**

1. Is the manuscript technically sound, and do the data support the conclusions?

The manuscript must describe a technically sound piece of scientific research with data that supports the conclusions. Experiments must have been conducted rigorously, with appropriate controls, replication, and sample sizes. The conclusions must be drawn appropriately based on the data presented.

Reviewer #1: Partly

Reviewer #2: Yes

**********

2. Has the statistical analysis been performed appropriately and rigorously?

Reviewer #1: Yes

Reviewer #2: Yes

**********

3. Have the authors made all data underlying the findings in their manuscript fully available?

The PLOS Data policy requires authors to make all data underlying the findings described in their manuscript fully available without restriction, with rare exception (please refer to the Data Availability Statement in the manuscript PDF file). The data should be provided as part of the manuscript or its supporting information, or deposited to a public repository. For example, in addition to summary statistics, the data points behind means, medians and variance measures should be available. If there are restrictions on publicly sharing data—e.g. participant privacy or use of data from a third party—those must be specified.

Reviewer #1: Yes

Reviewer #2: Yes

**********

4. Is the manuscript presented in an intelligible fashion and written in standard English?

PLOS ONE does not copyedit accepted manuscripts, so the language in submitted articles must be clear, correct, and unambiguous. Any typographical or grammatical errors should be corrected at revision, so please note any specific errors here.

Reviewer #1: Yes

Reviewer #2: Yes

**********

5. Review Comments to the Author

Please use the space provided to explain your answers to the questions above. You may also include additional comments for the author, including concerns about dual publication, research ethics, or publication ethics. (Please upload your review as an attachment if it exceeds 20,000 characters)

Reviewer #1: Review report April 17, 2026

The submitted manuscript entitled “The upper and lower airway microbiota and coronary heart disease in COPD patients

and controls” has discussed timely interesting topic, the connection between the respiratory microbiota and CHD patients. However, some weak points listed below make the publication of this article in its current status incompetent.

1- Microbiome is diversified subject and the exclusion and inclusion criteria considered in this study design are not satisfying.

2- COPD or CHD patients certainly receive non-antibiotic medicines which will directly or indirectly change host-microbiome interactions. Do all participants have same medication regimen?

3- Oral wash does not reflect the upper respiratory tract. It is rather reflective of oral microbiome, which is directly influenced by food and drink styles, general gum health or too many other factors.

4- Ignoring the roles of different bacterial communities in the discussion chapter weakens the scientific approach of this article. Furthermore, bioinformatics could have been better if the PICRUST analysis was added.

Reviewer #2: Possible association between airway microbiome and coronary atherosclerosis is an emerging research field and i see this study as a great effort to find any association. it is technically sound and presented in easy to understand fashion.

**********

6. PLOS authors have the option to publish the peer review history of their article (what does this mean?). If published, this will include your full peer review and any attached files.

If you choose “no”, your identity will remain anonymous but your review may still be made public.

**Do you want your identity to be public for this peer review?**  For information about this choice, including consent withdrawal, please see our Privacy Policy.

Reviewer #1: **Yes:**  Hasan Alghetaa

Reviewer #2: **Yes:**  Devendra Tripathi

**********

[NOTE: If reviewer comments were submitted as an attachment file, they will be attached to this email and accessible via the submission site. Please log into your account, locate the manuscript record, and check for the action link "View Attachments". If this link does not appear, there are no attachment files.]

To ensure your figures meet our technical requirements, please review our figure guidelines: https://journals.plos.org/plosone/s/figures

You may also use PLOS’s free figure tool, NAAS, to help you prepare publication quality figures: https://journals.plos.org/plosone/s/figures#loc-tools-for-figure-preparation.

NAAS will assess whether your figures meet our technical requirements by comparing each figure against our figure specifications.

# Author comment: Author response to Decision Letter 1

**DOI:** 10.1371/journal.pone.0353738.r002

16 May 2026

Dear Editor,

We thank you for the opportunity to revise our manuscript and provide a point-by-point response to the reviewers. We have tried to improve the manuscript, particularly considering the advice of the editor to clarify the study design and methods, ensure that the statistical approach and interpretation are fully justified, and align the conclusions closely with the data presented.

Note, we have in our response to reviewer 1 performed and present some analyses. They do not format well in the online submission form, however they are also uploaded the response to the reviewers as a separate new file, which is probably easier to read.

Reviewer #1: Review report April 17, 2026

The submitted manuscript entitled “The upper and lower airway microbiota and coronary heart disease in COPD patients

and controls” has discussed timely interesting topic, the connection between the respiratory microbiota and CHD patients. However, some weak points listed below make the publication of this article in its current status incompetent.

1- Microbiome is diversified subject and the exclusion and inclusion criteria considered in this study design are not satisfying.

We admit being a bit confused about this statement; that is, it is not inherently clear to us what is meant by "Microbiome is diversified subject". We have interpreted this statement to mean that the microbiome as a study term is a diverse topic, in that it encompasses many potential connections, ecological niches or habitats which may or may not impact health. This we certainly agree with. However, to link that with which inclusion or exclusion criteria are satisfying or not in a study on the potential association between the airways microbiome and cardiac health is not immediately obvious to us.

Our study is observational, and therefore by default vulnerable to selection bias. However, studying the lower airways microbiome by bronchoscopic sampling includes having a procedure performed which may mean some discomfort to the study subjects. In our cohort, all procedures were performed on volunteers, with no financial gain, or any other gain, as the bronchoscopies were not performed for any diagnostic purposes. This design avoids the bias of including patients being examined for a reason, thus being likely to have a disease characteristic which may impact the results, like an ongoing COPD exacerbation, pneumonia, or potentially lung cancer.

Now, although bronchoscopy is overwhelmingly a safe procedure, it is an invasive procedure with the potential for some side effects, notably A) bleeding (not from washings, but in some patients, we included bronchial brushes and bronchial biopsies. Also, we needed to be able to sample unexpected visual endobronchial changes, thus bleeding risk was always considered), B) inducing bronchospasm, C) allergy to procedural sedation, D) cardiac events like atrial fibrillation, or acute coronary events, and E) adverse reactions to alfentanil (respiratory depression). These effects are all rare, and mostly manageable in the bronchoscopy theatre, but of course, we took every precaution possible to avoid them. The meant that having a history of unstable coronary disease, atrial fibrillation, recent thromboembolic disease, use of anti-coagulants other than acetyl salicylic acid, and known allergy to any of the procedural medications were contraindications to participate.

To avoid bronchospasm which could happen in COPD (or asthma) patients; both patients and controls received a dose of salbutamol before the bronchoscopy (standard during the lung function test taken before the bronchoscopy), and they were only examined while in the stable state. Further, we avoided taking bronchoalveolar lavage if the lung function corresponded to an FEV1 less than 30% or 1 liter. As part of the MicroCOPD study we followed our patients closely for any discomfort, complications or side effects, and this has been published1.

Now, one could argue that this excludes unstable and potentially more severely heart disease afflicted COPD patients and controls, but such were the restraints from any ethical perspective. Also, the exclusion criteria above applied equally to all study participants. In addition to this, all adults in the study were > 40 years of age, a standard requirement for diagnosing COPD, which almost never starts before the age of 40 (in such cases it would usually be confounding asthma).

Another requirement was not having taken any antibiotic within the last 14 days (for any indication). Obviously, this was chosen to avoid impacting the microbiome. In retrospect, 14 days were perhaps a bit short, as some residual effect of antibiotics could be present after 15 days. We chose 14 days to avoid excluding an important group of COPD patients, namely those who frequently exacerbate, and therefore are more likely to have several courses of antibiotics taken during a year. However, in previous publications from the MicroCOPD study, other reviewers have pointed to these 14 days as perhaps being a source of impact on microbial profiling, and we therefore performed a journal review and analyzed use of antibiotics the last month, 3 months, and 12 months. This has been published before2,3. Briefly, 3% (6 subjects) had taken AB < 4 weeks before the bronchoscopy and 11% < 3 months before.

We did not have an upper age limit in the study and included both sexes. We allowed smokers and non-smokers (a smoking history of > 10 pack years was necessary in COPD, a standard requirement in most studies to avoid confounding with asthma), and recorded all medication use participants had, which we address in detail at point 2.

We take the reviewers' and editors' comments to mean that we should provide more details on inclusion and exclusion criteria in the current paper for increased clarity for the reader, and we have added some text to the methods section to address this.

2- COPD or CHD patients certainly receive non-antibiotic medicines which will directly or indirectly change host-microbiome interactions. Do all participants have same medication regimen?

As our controls were recruited from previous population cohorts, and COPD patients have many comorbidities, our study participants did indeed use a large range of non-antibiotic medications as the reviewer states.

Now, non-antibiotic medications may mean less than the reviewer believes. One medication we have been concerned about in particular, is inhaled corticosteroids (ICS), which is taken by several COPD patients as part of their medication regimen but is not taken by lung healthy controls. Corticosteroids are immunosuppressants, and oral thrash (mild local fungal disease usually by candida) is a common side effect. Thus, it would be logical that inhaled corticosteroids could impact both the oral and lower airways bacterial microbiome. Now, the study question in the current study was whether the oral or lower airways microbiome was associated with coronary heart disease. This we examined both in controls and COPD patients separately, and therefore in controls ICS would regardless not be a factor.

The effects of ICS in COPD we have examined in some detail in our previous studies. The effects on the fungal microbiome has been there4, but on the bacterial microbiome no difference in either diversity or taxonomy (differential abundance, DA) was seen between patients who used and those who did not use ICS3.

We performed both unadjusted and adjusted analyses on our study question, with reasonably similar results. We are pleased to know we have performed the largest single center study with bronchoscopy sampling of healthy adults to date, yet our sample size is still vulnerable to lack of strength if we include too many covariables. Knowing ICS did not impact the microbiome in our cohort we chose to exclude them here.

Perhaps more difficult is handling medications for heart disease. Obviously, many of our patients had known heart disease, and took medications as such. Of particular difficulty would be handling of statins since statin use could in theory elevate or mask significant calcium scores. However, the distribution of medication use in our study sample is actually reasonably even, considering the COPD patients in general are prone to have more comorbidities:

Controls (n=101) COPD patients (n=127)

Medication use n % n % p

Long acting muscarinic antagonists (LAMA) 0 0 79 62.2 <0.01

Long acting beta2 agonists 0 0 87 68.5 <0.01

Inhaled corticosteroids 0 0 80 63.0 <0.01

Angiotensin Converting Enzyme Inhibitors 2 2.0 3 2.4 0.85

Angiotensin Receptor Blockers 10 9.9 17 13.4 0.42

Any anti-hypertensive drug 18 17.8 29 22.8 0.35

Acetyl salicylic acid 13 12.9 34 26.7 0.01

Statin 21 20.8 37 29.1 0.15

Proton pump inhibitors 6 5.9 19 15.0 0.03

The most important questions are perhaps whether there are systematic differences between those with and without CHD in either controls or COPD patients:

In Controls only:

no sign CaSc Sign CaSc

Medication use n % n % p

Angiotensin Receptor Blockers 7 10.0 3 9.7 0.96

Any anti-hypertensive drug 11 15.7 7 22.6 0.41

Acetyl salicylic acid 5 7.1 8 25.8 0.01

Statin 6 8.6 15 48.4 <0.01

Proton pump inhibitors 4 5.7 2 6.5 0.60

In COPD only:

no sign CaSc Sign CaSc

Medication use n % n % p

Long acting muscarinic antagonists (LAMA) 30 51.7 48 71.6 0.02

Long acting beta2 agonists 37 63.8 49 73.1 0.26

Inhaled corticosteroids 34 58.6 45 67.2 0.32

Angiotensin Converting Enzyme Inhibitors 1 1.7 2 3.0 0.55

Angiotensin Receptor Blockers 6 10.3 9 13.4 0.60

Any anti-hypertensive drug 9 15.5 18 26.9 0.12

Acetyl salicylic acid 8 13.8 24 35.8 0.01

Statin 8 13.8 27 40.3 <0.01

Proton pump inhibitors 6 10.3 13 19.4 0.16

Unsurprisingly, having a high Calcium Score was consistent with higher use of statins and platelet inhibitors, as many subjects knew from before they had CHD.

Now, we have not come across a great deal of evidence that the oral or the lower airways microbiome is impacted by these medications.

In a Japanese study on 82 older subjects with "with a heterogeneous background of comorbidities". In that study, there was some evidence that salivary streptococcal abundance was a bit lower in subjects taking statins.

However, in a Canadian study published in PLoS One 2021 Dec 9;16(12):e0261032. doi: 10.1371/journal.pone.0261032), more than 1000 subjects had saliva samples profiled, and correlated with medication use. Although a few statistically DA taxa were found, the conclusion of the authors were:

"The results from this study show negligible effect of commonly used medications on microbial diversity and small differences in the relative abundance of specific taxa, suggesting a minimal influence of commonly used medication on the salivary microbiome of individuals living without major chronic conditions."

On the lower airways microbiome, we have found no previously published data.

As expected in a clinical cohort of COPD patients, participants used a range of medications including inhaled bronchodilators (LAMA/LABA), inhaled corticosteroids, statins, ACE inhibitors, ARBs, acetylsalicylic acid, and proton pump inhibitors. We have re-run all multivariable diversity and DA analyses, adding each (one by one to avoid collinearity and overstretching our regression models) of the medications listed in the tables above.

For alpha diversity (Shannon Index), the CaSc>100 effect estimate remained stable across all medication-adjusted models, with no medication changing the non-significant association (Table A):

Supplementary Table: Medication sensitivity analyses — effect of CaSc on alpha and beta diversity

Table A. Alpha diversity (Shannon index) — linear regression coefficient (β) for sign_CACS (p-value). Each row shows the effect of calcium score in alpha diversity when the row medication is added as a covariate.

Alpha diversity (Shannon index) — β for CaSc (p-value)

Unadjusted Adjusted† Unadjusted Adjusted† Unadjusted Adjusted† Unadjusted Adjusted†

Medication added OW · Controls (n = 101) OW · COPD (n = 121) BAL · Controls (n = 98) BAL · COPD (n = 95)

None (base model) 0.127 (p=0.121) 0.142 (p=0.136) -0.109 (p=0.239) -0.010 (p=0.922) 0.076 (p=0.658) 0.061 (p=0.758) -0.144 (p=0.404) -0.256 (p=0.176)

LAMA – – -0.093 (p=0.321) 0.010 (p=0.921) – – -0.153 (p=0.383) -0.274 (p=0.158)

LABA – – -0.101 (p=0.275) -0.007 (p=0.946) – – -0.144 (p=0.408) -0.257 (p=0.177)

Inhaled corticosteroid – – -0.102 (p=0.272) -0.007 (p=0.943) – – -0.138 (p=0.425) -0.255 (p=0.181)

ACE inhibitor 0.119 (p=0.147) 0.135 (p=0.156) -0.104 (p=0.267) -0.008 (p=0.941) 0.071 (p=0.680) 0.057 (p=0.775) -0.138 (p=0.424) -0.262 (p=0.168)

ARB 0.126 (p=0.118) 0.140 (p=0.135) -0.112 (p=0.226) -0.015 (p=0.886) 0.078 (p=0.647) 0.062 (p=0.752) -0.147 (p=0.396) -0.269 (p=0.158)

Antihypertensive 0.140 (p=0.085) 0.147 (p=0.117) -0.135 (p=0.144) -0.037 (p=0.716) 0.093 (p=0.588) 0.071 (p=0.719) -0.153 (p=0.381) -0.270 (p=0.158)

Acetylsalicylic acid 0.143 (p=0.092) 0.154 (p=0.114) -0.090 (p=0.352) 0.008 (p=0.939) 0.114 (p=0.525) 0.086 (p=0.672) -0.157 (p=0.386) -0.250 (p=0.197)

Statin 0.167 (p=0.070) 0.178 (p=0.084) -0.134 (p=0.168) -0.036 (p=0.737) 0.110 (p=0.569) 0.111 (p=0.605) -0.133 (p=0.468) -0.242 (p=0.218)

PPI 0.128 (p=0.120) 0.141 (p=0.140) -0.082 (p=0.364) 0.015 (p=0.883) 0.079 (p=0.642) 0.054 (p=0.783) -0.125 (p=0.474) -0.239 (p=0.211)

Beta diversity (Bray-Curtis PERMANOVA) showed no significant difference in community-level composition and with consistently small R2-values across all strata and all medication-adjusted models, indicating that community composition does not differ by CaSc status regardless of medication adjustment (Table B).

Table B. Beta diversity (Bray-Curtis, PERMANOVA) — marginal R² for CaSc (p-value). Each row shows the effect of calcium score when the row medication is added as a covariate.

Beta diversity (Bray-Curtis PERMANOVA) — R² for CaSc (p-value)

Unadjusted Adjusted† Unadjusted Adjusted† Unadjusted Adjusted† Unadjusted Adjusted†

Medication added OW · Controls (n = 101) OW · COPD (n = 121) BAL · Controls (n = 98) BAL · COPD (n = 95)

None (base model) 0.011 (p=0.286) 0.008 (p=0.671) 0.008 (p=0.403) 0.007 (p=0.547) 0.008 (p=0.820) 0.007 (p=0.917) 0.007 (p=0.876) 0.006 (p=0.962)

LAMA – – 0.008 (p=0.414) 0.008 (p=0.499) – – 0.007 (p=0.848) 0.006 (p=0.965)

LABA – – 0.008 (p=0.463) 0.007 (p=0.567) – – 0.007 (p=0.872) 0.006 (p=0.962)

Inhaled corticosteroid – – 0.008 (p=0.487) 0.007 (p=0.603) – – 0.007 (p=0.864) 0.006 (p=0.955)

ACE inhibitor 0.011 (p=0.299) 0.008 (p=0.599) 0.008 (p=0.381) 0.008 (p=0.522) 0.008 (p=0.822) 0.007 (p=0.927) 0.008 (p=0.834) 0.006 (p=0.954)

ARB 0.011 (p=0.321) 0.008 (p=0.664) 0.008 (p=0.412) 0.007 (p=0.589) 0.008 (p=0.792) 0.007 (p=0.917) 0.008 (p=0.855) 0.007 (p=0.945)

Antihypertensive 0.011 (p=0.254) 0.008 (p=0.677) 0.008 (p=0.392) 0.007 (p=0.593) 0.008 (p=0.783) 0.007 (p=0.912) 0.008 (p=0.833) 0.007 (p=0.938)

Acetylsalicylic acid 0.014 (p=0.160) 0.009 (p=0.490) 0.007 (p=0.577) 0.007 (p=0.637) 0.010 (p=0.440) 0.007 (p=0.821) 0.007 (p=0.889) 0.006 (p=0.965)

Statin 0.013 (p=0.200) 0.010 (p=0.416) 0.007 (p=0.689) 0.006 (p=0.804) 0.011 (p=0.290) 0.009 (p=0.648) 0.007 (p=0.902) 0.006 (p=0.967)

PPI 0.011 (p=0.293) 0.008 (p=0.647) 0.008 (p=0.416) 0.007 (p=0.568) 0.008 (p=0.807) 0.007 (p=0.904) 0.007 (p=0.871) 0.006 (p=0.972)

† Adjusted: age, sex, smoking status included as covariates in addition to the listed medication.

– Not applicable: medication class not used in this diagnostic group (e.g., LAMA/LABA/ICS absent in controls).

Base model (yellow): sign_CACS only (unadjusted) or sign_CACS + age + sex + smoking (adjusted), without any medication covariate.

All p-values are two-sided. None of the sign_CACS effects reached statistical significance in any model.

OW = oral wash; BAL = bronchoalveolar lavage; LAMA = long-acting muscarinic antagonist; LABA = long-acting β2-agonist; ICS = inhaled corticosteroid; ACE = angiotensin-converting enzyme; ARB = angiotensin receptor blocker; PPI = proton pump inhibitor.

Regarding differential abundance, we conducted similar sensitivity analyses. The base model represents the adjusted estimate (CaSc + age + sex + smoki

Attachment Submitted filename: Response to referee PLOS One May 2026.docx

# Editor report: Decision Letter 1

**DOI:** 10.1371/journal.pone.0353738.r003

**Authors:** Jamal Akhtar, Jamal Akhtar

27 May 2026

PONE-D-26-08565R1

The oral and lower airway microbiota and coronary heart disease in COPD patients and controls

PLOS One

Dear Dr. Svendsen,

Thank you for submitting your manuscript to PLOS ONE. After careful consideration, we feel that it has merit but does not fully meet PLOS ONE’s publication criteria as it currently stands. Therefore, we invite you to submit a revised version of the manuscript that addresses the points raised during the review process.

Please submit your revised manuscript by Jul 11 2026 11:59PM. If you will need more time than this to complete your revisions, please reply to this message or contact the journal office at plosone@plos.org. When you're ready to submit your revision, log on to https://www.editorialmanager.com/pone/ and select the 'Submissions Needing Revision' folder to locate your manuscript file.

Please include the following items when submitting your revised manuscript:

- A letter that responds to each point raised by the academic editor and reviewer(s). You should upload this letter as a separate file labeled 'Response to Reviewers'.
- A marked-up copy of your manuscript that highlights changes made to the original version. You should upload this as a separate file labeled 'Revised Manuscript with Track Changes'.
- An unmarked version of your revised paper without tracked changes. You should upload this as a separate file labeled 'Manuscript'.

If you would like to make changes to your financial disclosure, please include your updated statement in your cover letter. Guidelines for resubmitting your figure files are available below the reviewer comments at the end of this letter.

If applicable, we recommend that you deposit your laboratory protocols in protocols.io to enhance the reproducibility of your results. Protocols.io assigns your protocol its own identifier (DOI) so that it can be cited independently in the future. For instructions see: https://journals.plos.org/plosone/s/submission-guidelines#loc-laboratory-protocols. Additionally, PLOS ONE offers an option for publishing peer-reviewed Lab Protocol articles, which describe protocols hosted on protocols.io. Read more information on sharing protocols at https://plos.org/protocols?utm_medium=editorial-email&utm_source=authorletters&utm_campaign=protocols.

As the corresponding author, your ORCID iD is verified in the submission system and will appear in the published article. PLOS supports the use of ORCID, and we encourage all coauthors to register for an ORCID iD and use it as well. Please encourage your coauthors to verify their ORCID iD within the submission system before final acceptance, as unverified ORCID iDs will not appear in the published article. *Only*  the individual author can complete the verification step; PLOS staff *cannot*  verify ORCID iDs on behalf of authors.

We look forward to receiving your revised manuscript.

Kind regards,

Jamal Akhtar

Academic Editor

PLOS One

Journal Requirements:

If the reviewer comments include a recommendation to cite specific previously published works, please review and evaluate these publications to determine whether they are relevant and should be cited. There is no requirement to cite these works unless the editor has indicated otherwise.

Please review your reference list to ensure that it is complete and correct. If you have cited papers that have been retracted, please include the rationale for doing so in the manuscript text, or remove these references and replace them with relevant current references. Any changes to the reference list should be mentioned in the rebuttal letter that accompanies your revised manuscript. If you need to cite a retracted article, indicate the article’s retracted status in the References list and also include a citation and full reference for the retraction notice.

Additional Editor Comments:

Dear Dr. Svendsen and co-authors,

Thank you for submitting your carefully revised manuscript (PONE-D-26-08565R1) and your detailed point-by-point response to the reviewer comments. I have reviewed your response thoroughly and am pleased to note that you have addressed the major concerns raised in the previous round in a rigorous and scientifically sound manner. I am now requesting a Minor Revision before a final acceptance decision.

Below is a summary of my assessment of your responses:

1. INCLUSION/EXCLUSION CRITERIA (Reviewer 1, Point 1):

Your response clearly and comprehensively explains the study design rationale, including the volunteer-based bronchoscopy approach, the 14-day antibiotic washout period, the safety-driven exclusion criteria for bronchoscopy, and the age requirements. The transparency about the limitations of these design choices (e.g., the potential residual antibiotic effect after 14 days) is appreciated. The addition of expanded text in the Methods section is appropriate.

Remaining request: Please ensure that the updated Methods text in the revised manuscript explicitly lists the primary inclusion and exclusion criteria in a clear, structured manner so that readers can readily assess the study population without needing to refer to the supplementary publications.

2. MEDICATION CONFOUNDING (Reviewer 1, Point 2):

Your sensitivity analyses (Tables A-D) represent a substantial and commendable analytical addition to this work. The demonstration that the primary finding — the lack of a significant association between airway microbiota and coronary calcium score — remains robust after adjusting for each medication class individually is reassuring and strengthens the manuscript considerably. The observation that Lactobacillus enrichment in oral wash controls with high CaSc remains significant in most medication-adjusted models is also noteworthy.

Remaining request: Please ensure that the new sensitivity analysis tables are included as supplementary material in the revised manuscript, and that the Methods and Discussion sections clearly reference and describe these analyses.

3. ORAL WASH METHODOLOGY (Reviewer 1, Point 3):

Your decision to correct all references from "upper airways" to "oral" microbiome throughout the manuscript is appropriate and appreciated. This clarification meaningfully improves the accuracy of the manuscript.

Remaining request: Please confirm that all instances of "upper airway" have been updated, and that the Discussion acknowledges the scope and limitations of oral wash as a proxy for microbiome assessment in this context.

4. DISCUSSION OF BACTERIAL COMMUNITIES (Reviewer 1, Point 4 - Discussion):

Thank you for expanding the Discussion section to better address the roles of specific bacterial taxa identified. This is an important improvement that strengthens the scientific rigor of the article.

Remaining request: Please ensure that the expanded discussion specifically addresses the biologically plausible mechanisms linking the oral microbiome (particularly Lactobacillus enrichment in high CaSc controls) with cardiovascular health, as this is a key finding of the study.

5. PICRUST2 FUNCTIONAL ANALYSIS (Reviewer 1, Point 4 - Bioinformatics):

I commend you for conducting the PICRUSt2 analysis in response to the reviewer's suggestion. Your finding that no significant functional pathway differences were detected between high and low calcium score groups is an important negative result. Your explanation of the inherent limitations of 16S-based functional inference at genus level (e.g., the Lactobacillus/Clostridium and Corynebacterium/Mycobacterium examples) is scientifically sound and provides appropriate caution.

The decision not to include PICRUSt2 results in the main manuscript is acceptable given the null findings and methodological limitations. However, please include the PICRUSt2 results as a supplementary table, as this adds transparency and will be valuable to readers interested in the functional microbiome aspects.

In summary, this revised manuscript represents a substantial improvement over the original submission, and the authors' responses demonstrate a thorough and scientifically rigorous approach to addressing the reviewers' concerns. With the minor revisions requested above, I anticipate this manuscript will be suitable for acceptance.

Please submit your revised manuscript along with a point-by-point response to the items listed above.

Sincerely,

Jamal Akhtar, MD

Academic Editor, PLOS ONE

[Note: HTML markup is below. Please do not edit.]

[NOTE: If reviewer comments were submitted as an attachment file, they will be attached to this email and accessible via the submission site. Please log into your account, locate the manuscript record, and check for the action link "View Attachments". If this link does not appear, there are no attachment files.]

To ensure your figures meet our technical requirements, please review our figure guidelines: https://journals.plos.org/plosone/s/figures

You may also use PLOS’s free figure tool, NAAS, to help you prepare publication quality figures: https://journals.plos.org/plosone/s/figures#loc-tools-for-figure-preparation.

NAAS will assess whether your figures meet our technical requirements by comparing each figure against our figure specifications.

# Author comment: Author response to Decision Letter 2

**DOI:** 10.1371/journal.pone.0353738.r004

1 Jun 2026

Dear Editor,

Thank you for the opportunity to further revise our manuscript and provide a more detailed response to the reviewers.

1. INCLUSION/EXCLUSION CRITERIA (Reviewer 1, Point 1):

Your response clearly and comprehensively explains the study design rationale, including the volunteer-based bronchoscopy approach, the 14-day antibiotic washout period, the safety-driven exclusion criteria for bronchoscopy, and the age requirements. The transparency about the limitations of these design choices (e.g., the potential residual antibiotic effect after 14 days) is appreciated. The addition of expanded text in the Methods section is appropriate.

Remaining request: Please ensure that the updated Methods text in the revised manuscript explicitly lists the primary inclusion and exclusion criteria in a clear, structured manner so that readers can readily assess the study population without needing to refer to the supplementary publications.

We thank the Editor for this request and have further detailed the methods section to include a structured presentation of inclusion and exclusion criteria. The revised text will hopefully allow readers to assess the study population without reference to supplementary publications.

2. MEDICATION CONFOUNDING (Reviewer 1, Point 2):

Your sensitivity analyses (Tables A-D) represent a substantial and commendable analytical addition to this work. The demonstration that the primary finding — the lack of a significant association between airway microbiota and coronary calcium score — remains robust after adjusting for each medication class individually is reassuring and strengthens the manuscript considerably. The observation that Lactobacillus enrichment in oral wash controls with high CaSc remains significant in most medication-adjusted models is also noteworthy.

Remaining request: Please ensure that the new sensitivity analysis tables are included as supplementary material in the revised manuscript, and that the Methods and Discussion sections clearly reference and describe these analyses.

We thank the Editor for this assessment. The sensitivity analysis tables have been included as Supplementary S2-S5 Tables in the revised manuscript. The methods and discussion sections have been updated as follows.

3. ORAL WASH METHODOLOGY (Reviewer 1, Point 3):

Your decision to correct all references from "upper airways" to "oral" microbiome throughout the manuscript is appropriate and appreciated. This clarification meaningfully improves the accuracy of the manuscript.

Remaining request: Please confirm that all instances of "upper airway" have been updated, and that the Discussion acknowledges the scope and limitations of oral wash as a proxy for microbiome assessment in this context.

We confirm that all instances of “upper airway microbiome” have been replaced with “oral microbiome” throughout the manuscript and added it as a limitation.

4. DISCUSSION OF BACTERIAL COMMUNITIES (Reviewer 1, Point 4 - Discussion):

Thank you for expanding the Discussion section to better address the roles of specific bacterial taxa identified. This is an important improvement that strengthens the scientific rigor of the article.

Remaining request: Please ensure that the expanded discussion specifically addresses the biologically plausible mechanisms linking the oral microbiome (particularly Lactobacillus enrichment in high CaSc controls) with cardiovascular health, as this is a key finding of the study.

We thank the Editor for this request. The discussion section has been expanded to address the plausible mechanisms linking the oral microbiome to coronary heart disease.

5. PICRUST2 FUNCTIONAL ANALYSIS (Reviewer 1, Point 4 - Bioinformatics):

I commend you for conducting the PICRUSt2 analysis in response to the reviewer's suggestion. Your finding that no significant functional pathway differences were detected between high and low calcium score groups is an important negative result. Your explanation of the inherent limitations of 16S-based functional inference at genus level (e.g., the Lactobacillus/Clostridium and Corynebacterium/Mycobacterium examples) is scientifically sound and provides appropriate caution.

The decision not to include PICRUSt2 results in the main manuscript is acceptable given the null findings and methodological limitations. However, please include the PICRUSt2 results as a supplementary table, as this adds transparency and will be valuable to readers interested in the functional microbiome aspects.

We thank the editor for this request. PICRUSt2 results are now included in the discussion and added as Supplementary S3-S4 Figure.

Attachment Submitted filename: Response to Editor_ONE-D-26-08565_29may26.docx

# Editor report: Decision Letter 2

**DOI:** 10.1371/journal.pone.0353738.r005

**Authors:** Jamal Akhtar, Jamal Akhtar, Jamal Akhtar

29 Jun 2026

The oral and lower airway microbiota and coronary heart disease in COPD patients and controls

PONE-D-26-08565R2

Dear Dr. Svendsen,

We’re pleased to inform you that your manuscript has been judged scientifically suitable for publication and will be formally accepted for publication once it meets all outstanding technical requirements.

Within one week, you’ll receive an e-mail detailing the required amendments. When these have been addressed, you’ll receive a formal acceptance letter and your manuscript will be scheduled for publication.

An invoice will be generated when your article is formally accepted. Please note, if your institution has a publishing partnership with PLOS and your article meets the relevant criteria, all or part of your publication costs will be covered. Please make sure your user information is up-to-date by logging into Editorial Manager at Editorial Manager® and clicking the ‘Update My Information' link at the top of the page. For questions related to billing, please contact billing support.

If your institution or institutions have a press office, please notify them about your upcoming paper to help maximize its impact. If they’ll be preparing press materials, please inform our press team as soon as possible -- no later than 48 hours after receiving the formal acceptance. Your manuscript will remain under strict press embargo until 2 pm Eastern Time on the date of publication. For more information, please contact onepress@plos.org.

Kind regards,

Jamal Akhtar

Academic Editor

PLOS One

Additional Editor Comments (optional):

Reviewers' comments:

# Editor report: Acceptance letter

**DOI:** 10.1371/journal.pone.0353738.r006

**Authors:** Jamal Akhtar, Jamal Akhtar, Jamal Akhtar

PONE-D-26-08565R2

PLOS One

Dear Dr. Svendsen,

I'm pleased to inform you that your manuscript has been deemed suitable for publication in PLOS One. Congratulations! Your manuscript is now being handed over to our production team.

At this stage, our production department will prepare your paper for publication. This includes ensuring the following:

* All references, tables, and figures are properly cited

* All relevant supporting information is included in the manuscript submission,

* There are no issues that prevent the paper from being properly typeset

You will receive further instructions from the production team, including instructions on how to review your proof when it is ready. Please keep in mind that we are working through a large volume of accepted articles, so please give us a few days to review your paper and let you know the next and final steps.

Lastly, if your institution or institutions have a press office, please let them know about your upcoming paper now to help maximize its impact. If they'll be preparing press materials, please inform our press team within the next 48 hours. Your manuscript will remain under strict press embargo until 2 pm Eastern Time on the date of publication. For more information, please contact onepress@plos.org.

You will receive an invoice from PLOS for your publication fee after your manuscript has reached the completed accept phase. If you receive an email requesting payment before acceptance or for any other service, this may be a phishing scheme. Learn how to identify phishing emails and protect your accounts at https://explore.plos.org/phishing.

If we can help with anything else, please email us at customercare@plos.org.

Thank you for submitting your work to PLOS ONE and supporting open access.

Kind regards,

PLOS ONE Editorial Office Staff

on behalf of

Dr. Jamal Akhtar

Academic Editor

PLOS One
